# Unconventional fractional quantum Hall effect in bilayer graphene

**DOI:** 10.1038/s41598-017-09166-5

**Published:** 2017-08-18

**Authors:** Janusz Edward Jacak

**Affiliations:** Department of Quantum Technologies, Faculty of Fundamental Problems of Technology, Wrocław University of Science and Technology, Wyb. Wyspianskiego 27, 50-370 Wrocław, Poland

## Abstract

Recent experimental progress in Hall measurements in bilayer graphene in the so-called open-face configuration of boron nitride encapsulated samples, together with the earlier technique of suspended samples, allows for precise observation of the fractional quantum Hall effect (FQHE) in all 4 subbands of the Lowest Landau level (with *n* = 0 and *n* = 1) and in the next LL subbands (with *n* = 2) in the bilayer system. Many newly observed FQHE features do not agree with a conventional model of composite fermions and reveal a different hierarchy in comparison to monolayer graphene or GaAs 2DEG. We explain the peculiarity of the FQHE hierarchy in the bilayer system in the framework of a topological approach, which includes the composite fermion model as its special case. Inclusion of a topological effect caused by the hopping of electrons between the two sheets in the bilayer system allowed for an explanation of the FQHE hierarchy in the graphene bilayer in satisfactory accordance with the experimental observations.

## Introduction

The fractional quantum Hall effect (FQHE) is one of the most spectacular and mysterious quantum phenomena in condensed matter. Despite intensive experimental and theoretical studies of FQHE since the 80 s of the last century, the effect still eludes complete understanding. The FQHE apparently exceeds the conventional framework of local quantum mechanics and displays a trade-off between the Coulomb interaction induced localization and disorder^1^. It is commonly acknowledged that in the induced by the interaction formation of nonlocal specific correlations in FQHE, the central role is played by an exceptional topology of the planar continuum. An examination of FQHE in graphene is especially challenging. Graphene is perfectly two dimensional but with a pseudo-relativistic band structure. The band electron dynamics in graphene are dominated by the Dirac-like points in the corners of the hexagonal Brillouin zone, where the locally conical-shaped valence band meets the similarly conical-shaped conduction band, resulting in a linear-in-momentum local Hamiltonian^2^. This leads to non-equidistant LLs structure in the graphene monolayer, with energy proportional to $\sqrt{n}$ (*n* is number of the LL) instead of the ~*n* dependence in conventional 2DEG. In bilayer graphene, an interlayer tunnelling of electrons restores the parabolic local energy and almost-equidistant LLs, $\sim \sqrt{n(n-\mathrm{1)}}$ ^3^. The specific graphene pseudo-relativistic band structure close to the Dirac points, however, does not influence the topological constraints imposed on trajectories (upon the path-integral quantization), because the band structure is induced by a local electric interaction-type crystal field, which does not perturb the path topology, and the FQHE is observed in graphene, similarly to conventional semiconductor 2DEG systems.

In recent years, advances in the manufacturing methods of monolayer and bilayer ultra-clean graphene samples accelerated the development of the Hall experiment in this material, and several new observations of FQHE have been reported. Remarkably, the observations of FQHE in bilayer graphene^4–8^ reveal significant distinctions from FQHE manifestation in the graphene monolayer^9–12^. This is in conflict with a conventional imagination that FQHE is a manifestation of hypothetical composite fermions (CFs)^13^, i.e., electrons dressed with even number of localized on particles flux quanta of some auxiliary magnetic field. According to CF theory, such effective quasiparticles should be present both in monolayer and bilayer Hall systems, what however, does not agree with experimental observations in bilayer graphene^4–8^.

Important progress in the Hall experiment in graphene has been achieved by the invention of a measurement technique for suspended graphene small scrapings (both monolayer and bilayer)^4–8,11,12^ and by the mastering of an independent method for reducing substrate perturbations of graphene layers in samples supported by hexagonal boron nitride (hBN) crystal substrate^7,9,10^. The absence of a substrate (for suspended samples) or the avoidance of lattice mismatch (for hBN substrate) favour the delicate correlations induced by the interaction of 2D electrons, resulting in FQHE manifestation. The triggering role for FQHE organization is played by high electron mobility, well exceeding 100,000 cm^2^/(Vs) in both abovementioned experimental setups for Hall measurement in graphene.

A graphene sheet has regular planar hexagonal crystalline structure with two equivalent sublattices (two carbon atoms in the Bravais cell). This feature, together with the vanishing of the forbidden semiconductor gap at Dirac points, results in the four-fold spin-valley degeneracy of LLs in graphene monolayer^2^. Moreover, for Dirac points, the specific Berry phase-like shift for chiral 2D carriers additionally influences the LL spectrum, which finally results in the $\nu =4(n+\frac{1}{2})$ series for fillings at which the integral quantum Hall effect (IQHE) plateaus manifest themselves in monolayer graphene^2^. These plateaus in the monolayer graphene occur at the centres of the consecutive LLs (not completely filled LLs)^14^. In bilayer graphene, an extra degeneracy of the *n* = 0 and *n* = 1 oscillatory states in the LLL occurs and the twice as large Berry phase for chiral carriers shifts the IQHE plateau positions to the edges of LLs^3^. LLs in bilayer graphene are also four-fold spin-valley degenerate, except for the eight-fold degenerate lowest LL (LLL) (due to *n* = 0 and *n* = 1 degeneracy in the LLL)^2,14,15^. In the case of monolayer graphene, FQHE features are observed in the first six subbands of LLs with *n* = 0 and *n* = 1^9–12^, which reproduce a hierarchy similar to that in conventional semiconductor 2DEG. In bilayer graphene, observation of FQHE reaches even subbands with *n* = 2, revealing a different and unexpected FQHE hierarchy there^5,10^. Especially interesting are observations of unusual even-denominator fillings for FQHE in bilayer graphene in the LLL, including the most pronounced feature at $\nu =-\frac{1}{2}$ ^4^, which does not find any counterpart in monolayer systems. In particular, this state cannot be explained with the CF approach, as for CFs the Hall metal state is predicted at $\pm \frac{1}{2}$ ^13^.

In the present paper, we summarize the recent and controversial experimental observations of FQHE in bilayer graphene and compare them with the data for graphene monolayer. We identify the specific topological features that can explain the oddness of the correlated multi-particle states in the bilayer system in accordance with experimental observations. We propose an explanation for the exotic even-denominator fractions for FQHE in the LLL and the whole FQHE hierarchy in bilayer graphene in the framework of the topological commensurability approach^16–18^. Within this topological nonlocal braid group approach, we explain the structure of fractional fillings of LL subbands in accordance with experimental data and explain the reason for the insufficiency of the CF model in the bilayer graphene case. The topological braid group approach, formerly developed in refs^19,20^, gives the hierarchy of FQHE in agreement with the available experimental data for monolayer Hall systems and can be extended to the bilayer graphene case via identification of topological differences between mono and bilayer situations.

## Hierarchy of FQHE in graphene

The massive degeneracy of each LL subband in graphene is the same as in the conventional 2DEG, despite the different LL structure^2,3^, and is equal to $\frac{BS}{hc/e}$ (where *B* is the external magnetic field, *S* is the sample surface, and $\frac{hc}{e}$ is the magnetic field flux quantum). Nevertheless, the number of subbands per LL in graphene is different than in a conventional semiconductor case and equals 4 in graphene; it corresponds to the Zeeman spin splitting and to the valley pseudo-spin splitting (absent in conventional semiconductors) due to the mixing of two inequivalent Dirac points with two sublattices in graphene crystal lattice^2^. The Zeeman splitting in graphene is small^21^ and the valley splitting is small as well^2^, which results in the 4-fold approximate spin-valley degeneracy (referred to as SU(4) band symmetry). The LLL subbands are divided between particles and holes from the conduction and valence bands^2^. Hence, the bottom of the LLL is shifted upward by 2 (in terms of the filling factor). Conventionally, filling rates for holes from the valence band are assigned as negative numbers and are mirror reflections of the positive numbers denoting filling rates for electrons in the conduction band. An additional opportunity in graphene, beyond the ability of conventional 2DEG, is a possible control over the transition between particles and holes by the shifting of the Fermi level passing the Dirac point. Experimentally, it is realized by application of a relatively small lateral voltage (up to several dozen V), which determines the filling rate independently of the magnetic field strength.

Due to the interlayer electron hopping the local Hamiltonian for bilayer graphene re-attains the quadratic form with respect to the momentum. Hence, the LL spectrum in bilayer graphene resembles that of the ordinary 2DEG, but with four subbands for each LL level except for the LLL, which has eight-fold degeneracy^2,3^. As usual in graphene, the division of the LLL subbands equally between particles and holes causes the bottom, for uniformly charged carriers (electrons or holes), to be located in the centre of the 8-fold degenerate LLL. This extra degeneracy of the LLL is caused by the vanishing of energy of both the *n* = 0 and *n* = 1 oscillatory Landau states in the bilayer graphene, in contrast to the monolayer one. In the bilayer graphene, the Berry phase shift for chiral particles is also different (twice as large) in comparison to the graphene monolayer and is equal to 2*π* ^2^. Hence, the consecutive plateaus of IQHE are located in bilayer graphene at integer filling rates, whereas in monolayer graphene were located at half-fillings of LLs^2,3^.

### FQHE hierarchy in monolayer graphene

When the Fermi level is shifted (by the lateral voltage) to the conduction band and the magnetic field is strong enough that $\nu \in (0,1]$, we address the fractionally filled first conduction subband of the electron LLL, denoted as $n=0,2\uparrow$ (in this notation, $2$ denotes the valley pseudospin component and the arrow ↑ indicates the orientation of the ordinary spin along the magnetic field). For *N* < *N*_0_, the filling rate, *v* = *N*/*N*_0_, is fractional (the degeneracy *N*_0_ of each subband is ${N}_{0}=\frac{BS}{hc/e}$).

To decipher the FQHE hierarchy in this subband in graphene monolayer, we apply the braid group topological approach developed for the ordinary 2DEG system^17,22^. To implement braid group generators, the cyclotron orbit must be commensurate with the interparticle separation (the details are presented in SI). An archetype of the commensurability is $\frac{S}{N}=\frac{hc}{e{B}_{0}}=\frac{S}{{N}_{0}}$ (where *S* is the sample surface, *N* is the number of electrons, and *N*_0_ is LL degeneracy), as for $\nu =\frac{N}{{N}_{0}}=1$ and IQHE. Various more complicated patterns of the commensurability (cf. SI) define filling fractions for FQHE^17,22^.

In the case of graphene, an important property follows from the fact that cyclotron orbits in graphene are defined by the bare kinetic energy $T=\hslash {\omega }_{c}(n+\frac{1}{2})$ with ${\omega }_{c}=\frac{eB}{mc}$, similarly to the conventional semiconductor 2DEG (as in non-interacting 2D gas), despite the different pseudo-relativistic version of Landau-level energy. This is because the’relativistic’ oddness is caused by the peculiar crystal field (electric interaction of ions and electrons), which does not change the bare kinetic part of the Landau energy. Hence, the size of the braid cyclotron orbits for graphene is equal to the corresponding orbit size from the non-interacting gas.

Therefore, the cyclotron orbit size in the subband $n=0,2\uparrow$ is equal to $\frac{hc/e}{B}=\frac{S}{{N}_{0}}$. Because this orbit size is lower than the interparticle spacing expressed by $\frac{S}{N}$ (as $N < {N}_{0}$), multi-loop braids with enhanced size are needed to match neighbouring particles^17,22^ (cf. also SI for more detailed explanation). The commensurability condition, in this case, is as follows: $q\frac{S}{{N}_{0}}=\frac{S}{N}$, which gives $\nu =\frac{N}{{N}_{0}}=\frac{1}{q}$, (where *q* is an odd integer^17^). For the LL subband holes, the particle-hole symmetric filling rates $\nu =1-\frac{1}{q}$ are expected.

The next possible commensurability occurs when the last loop of the multi-loop cyclotron orbit is commensurate with every *l*th particle separation (similarly to in *l*th LL), whereas the *q* − 1 antecedent loops take away an integer number of flux quanta (which are commensurate with nearest-neighbouring particles). For $l=2,3,\ldots$, the last loop reaches every *l*th particles (next neighbours). In this manner, we obtain the hierarchy of fillings for FQHE in this LLL subband in the following form (the same as for the CF model): $\nu =\frac{l}{l(q-1)\pm 1},\quad \nu =1-\frac{l}{l(q-1)\pm 1}$, where $l=1,2,\ldots$ and the minus sign in the denominator, corresponds to the possibility of the reverse eight-figure orientation of the last loop with respect to the antecedent loop in the multi-loop orbit. Additionally, we notice that the filling rates for Hall metal states can be achieved in the limit $l\to \infty$ in the above formula, which corresponds to the situation in which the residual flux passing through the last loop tends to zero. This means that in such a case, the last loop can reach the infinitely distant particles as for fermions at the absence of a magnetic field, which is referred to the case of the Hall metal archetype for $\nu =\frac{1}{2}$ in the conventional 2DEG. Thus, in the limit $l\to \infty$, we arrive at the hierarchy for the Hall metal states in the form $\nu =\frac{1}{q-1},\quad \nu =1-\frac{1}{q-1}$.

Let us notice that the hierarchy of FQHE filling factors in the LLL of monolayer graphene is similar to the FQHE hierarchy in the conventional 2DEG (GaAs), due to the same commensurability structure in both cases. The role of the commensurability in identification of the FQHE filling factor hierarchy has been noted earlier^23^, inspired by its fractal-like character and by the Hofstadter’s butterfly picture^24^, on the other hand. The latter displays the commensurability of 2D cyclotron orbits with the crystalline cell, what, however, requires giant magnetic fields (~10^5^ T), which are out of reach in the experiment^24^. The commensurability with larger spatial scale, which in case of a diluted Wigner lattice is comparable with the magnetic length, ${l}_{B}=\sqrt{\frac{\hslash c}{eB}}$, may happen at lower magnetic fields, by four orders of magnitude, and can reproduce the fractal-like structure of FQHE filling factors, provided that the commensurability is defined under the braid group scheme, including multi-loop orbits.

One can also observe that other variants of commensurability may concern multi-loop orbits. Namely, each loop of the multi-loop structure may in principle be adjusted to particle separation in a different and mutually independent manner, matching nearest or next-nearest neighbours under various schemes. One such possibility may correspond to the situation in which *q*-loop orbit *q* − 1 loops are adjusted to every *x*th particle ($x=1,2,3,\ldots$), whereas the last one fits with every *l* ≠ *x*th particle separation. This commensurability scheme is observed in ordinary 2DEG Hall systems within the LLL for some exotic fractions, e.g., $\nu =\frac{4}{11},\frac{5}{13},\frac{3}{8},\frac{3}{10},\ldots$ (beyond the CF hierarchy corresponding only to *x* = 1, as detailed in SI). It is noticeable, however, that this series of exotic FQHE filling fractions have not yet been observed in the LLL in graphene, though they are observed in the first LL in monolayer graphene (as will be discussed below).

For lower magnetic fields, the next subband, the last one in the LLL, $n=0,2\downarrow$, is gradually filled with electrons. In this subband, the cyclotron orbit size $\frac{S}{{N}_{0}}$ is still lower than the interparticle separation $\frac{S}{N-{N}_{0}}$ (because $N-{N}_{0} < {N}_{0}$), similarly as in the antecedent subband, which causes repeating of the FQHE filling structure from the previous subband, but shifted ahead by 1. After complete filling of this subband, the LLL is completely filled as well. This gives the IQHE according to its main-line hierarchy, $\nu =4(n+\frac{1}{2})$, at *n* = 0.

In an analogous way, one can consider fillings of the following LLs. The nearest one corresponds to *n* = 1. This level has four, electron type subbands. The bare kinetic energy in this LL (in all its subbands) is equal to $\frac{3\hslash {\omega }_{c}}{2}$. In this subband, the cyclotron orbits are thus of size $\frac{3S}{{N}_{0}}$ (larger in comparison to the LLL) and they must be adjusted to the interparticle separation between electrons in this subband, $\frac{S}{N-2{N}_{0}}$. For a small number of electrons in the subband (close to the subband edge), we may have to deal with the multi-loop orbits if the single-loop orbits are too short, $\frac{3S}{{N}_{0}} < \frac{S}{N-2{N}_{0}}$. The *q*-loop orbits satisfy the commensurability condition $q\frac{3S}{{N}_{0}}=\frac{S}{N-2{N}_{0}}$, where *q* is an odd integer, which defines the main series for FQHE (multi-loop) in this subband, $\nu =2+\frac{1}{3q}$, however, shifted towards the subband edge in comparison to the LLL subband case. This main filling-line can be complemented to the complete related hierarchy, $\nu =2+\frac{l}{l3(q-1)\pm 1},\,\,\nu =3-\frac{l}{l3(q-1)\pm 1}\,l=i/3,\,i=1,2,\ldots$, with the Hall metal hierarchy in the limit $l\to \infty$, like that described in the case of the LLL. These series of filling rates are located closer to the subband edges in comparison to the FQHE rates in the LLL due to larger size of cyclotron orbits in the *n* = 1 LL subband.

Simultaneously, in the central part of this higher LL subband, a new type of commensurability is possible, which is not accessible in the LLL. This new commensurability occurs when $\frac{3S}{{N}_{0}}=\frac{xS}{N-2{N}_{0}}$ and *x* = 1, 2, 3, i.e., when the cyclotron orbit size exceeds the particle separation. Then, the single-loop orbit (large enough in this subband) can fit with every *x*th particle (*x*-order next-nearest neighbours). From this new commensurability opportunity, one finds fractions $\nu =\frac{7}{3},\frac{8}{3},3$ corresponding to single-loop cyclotron orbits (similarly to IQHE). Thus, for $\nu =\frac{7}{3},\frac{8}{3}$, we address the FQHE (single-loop). This is a new Hall feature that manifests only in higher LLs, where cyclotron orbits may be larger than the interparticle separation and single-loop orbits can reach next-nearest neighbours.

Let us note that the special case of the commensurability, $\frac{3S}{{N}_{0}}=\frac{1.5S}{N-2{N}_{0}}$, can be identified at $\nu =\frac{5}{2}$. This commensurability concerns the paired particles, rather than the single ones. The pairing does not change the cyclotron radius (being invariant upon doubling of mass and charge), but reduces by half the carrier number $\frac{N-2{N}_{0}}{2}$, which gives the above commensurability for pairs at $\nu =\frac{5}{2}$. Hence, at this filling rate, one can expect a manifestation of IQHE-type correlation, but for paired electrons (the considered correlation corresponds to *p*-like pairing due to the spin polarization in this subband).

**Figure 1:**
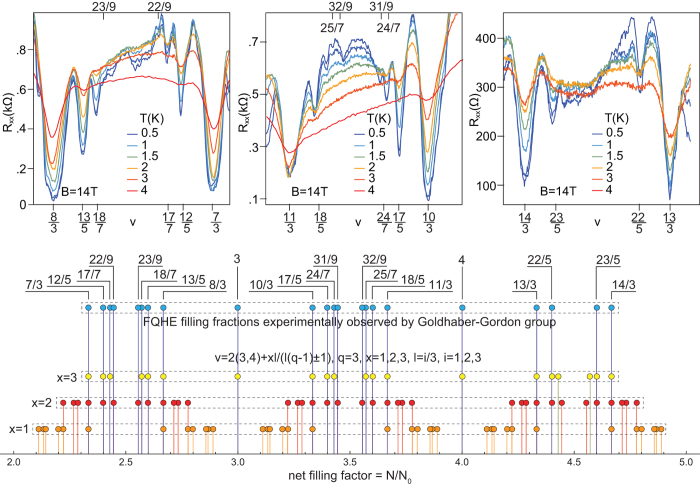
Fitting to experimental data of cyclotron braid hierarchy for FQHE in monolayer graphene in the first three subbands in the first LL (*n* = 1), $\nu \in (2,5]$. Upper panel—*R*_*xx*_ after experiment^10^, lower panel—the theoretical hierarchy. The larger residual longitudinal resistance (in the upper panel for $\frac{12}{5},\frac{17}{7},\frac{22}{9}$ and for other fractions with denominators 5, 7, 9) corresponds to correlated states of next-nearest electrons, of every second (*x* = 2) or every third (*x* = 3) particle, according to the commensurability series $\nu =2(3,4)+\frac{xl}{l3(q-1)\pm 1}$ with $q=3,\,x=2,3,\,l=\frac{i}{3},\,i=1,2,3$ (lower panel) (*x* = 1 corresponds to CF-like commensurability)—uncorrelated electrons enhance resistance.

A similar scheme of commensurability may be applied to the following subbands with *n* = 1. Moreover, an interesting new possibility for commensurability occurs for *q*-loop orbits with next-nearest neighbours. The sizes of particular loops in the multi-loop structure may, in general, be adjusted to the interparticle spacing in an independent way, resulting in new filling rates. In particular, this results in the hierarchy $\nu =2(3,4,5)+\frac{xl}{l3(q-1)\pm 1}$, $\nu =3(4,5,6)-\frac{xl}{l3(q-1)\pm 1}$ in all subbands of the first LL, which for $q=3,\,x=2,3,\,l=i/3,\,i=1,2,3$ reproduces $\nu =\frac{7}{3},\frac{8}{3}$, $\frac{12}{5},\frac{13}{5}$, $\frac{17}{7},\frac{18}{7}$, $\frac{22}{9},\frac{23}{9}$, $\frac{10}{3},\frac{11}{3}$, $\frac{17}{5},\frac{18}{5}$, $\frac{24}{7},\frac{25}{7}$, $\frac{13}{3},\frac{14}{3}$, $\frac{22}{5},\frac{23}{5}$. This opportunity for FQHE well agrees with the recent observations of FQHE in the first three subbands of the *n* = 1 LL in monolayer graphene at ultra-low temperatures^10^—cf. Fig. 1.

### FQHE hierarchy in bilayer graphene

**Figure 2:**
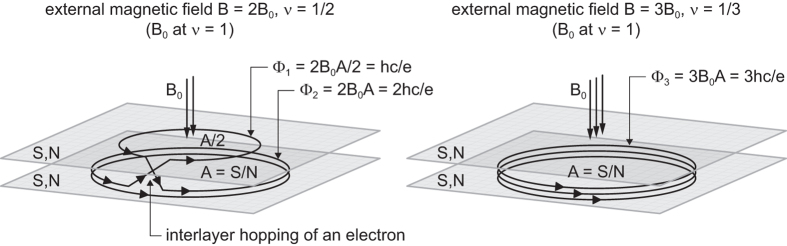
In the bilayer system, there are two possible topologically non-equivalent types of three-loop cyclotron trajectories (corresponding to particle exchange along the braid generator with one additional loop, ${\sigma }_{j}^{3}$, built from half of the 3-loop cyclotron orbits^22^). In the left panel, the three-loop orbit is distributed between two sheets—both sheets contribute their own magnetic fluxes, in contrast to the case when the three-loop orbit is located in a single sheet (right panel). This leads to the different commensurabilities in the following two situations. If loops are distributed between both layers, only two loops participate in the increasing of the orbit size, which gives the commensurability condition $A=\frac{S}{N}=2\frac{S}{{N}_{0}},\,{N}_{0}=\frac{BSe}{hc}\to \nu =\frac{1}{2}$. In the case when all three loops are placed in a single layer, the commensurability repeats that from the monolayer case and $\nu =\frac{1}{3}$.

In bilayer graphene, the topology of braid trajectories changes considerably in comparison to the monolayer system. The bilayer graphene is not strictly two dimensional and this opens a new possibility for the topology of the trajectories, as illustrated in Fig. 2.

Two sheets of the bilayer graphene lie at a close distance and electrons can hop between them. Multi-loop cyclotron orbits (and related braids) may thus reside in both layers simultaneously, i.e., loops may be distributed among both sheets. This makes a difference in comparison to the monolayer case because each sheet contributes to the total flux of the external magnetic field independently with its own surface, which strongly affects the cyclotron orbit size and braid commensurability condition. (cf. Fig. 2).

The simplest commensurability instance in the LLL (subband $n=0,2\uparrow$) with 3-loop cyclotron orbit located in both sheets of bilayer graphene (as illustrated in Fig. 2) results in filling fraction $\nu =\frac{1}{2}$, not $\frac{1}{3}$ as in the monolayer case. This exceptional fraction is observed experimentally (actually for holes at $\nu =-\frac{1}{2}$)^4^ and cannot be explained by the CF model (the CF model predicts a Hall metal state at $\nu =\pm \frac{1}{2}$).

**Figure 3:**
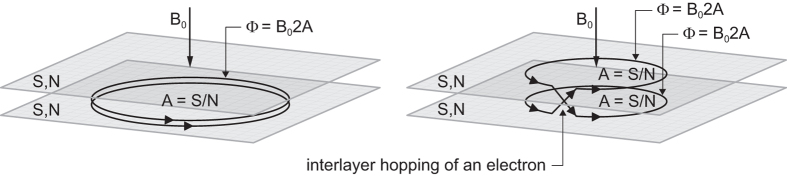
If a 2-loop orbit is distributed among two sheets (right), then the sizes of both loops are the same as that of a single loop (in the figure, *A* = *S*/*N*, as for *v* = 1 at *B*_0_), but if both loops are placed in a single sheet, the double planar orbit is twice as large (left). This results in different commensurabilities in those two situations at the same magnetic field.

The fact that the second loop of any pair of loops may be located in the opposite sheet of bilayer graphene with respect to the first loop—cf. Fig. 3—is the source of an oddness of FQHE hierarchy in bilayer graphene.

In general, in a bilayer system, loops of a multi-loop orbit may be located partly in both 2D sheets. To account for this effect in topological terms, adjusted to braid trajectories and commensurability requirements, one must neglect the contribution of a single loop in the multi-loop structure when the total flux of the external field is divided into fractions per loop. This single loop captures its own flux, whereas the remaining loops will share an identical flux—the one which passes through any cyclotron orbit in the monolayer case. Removal of the single loop must be performed independently of how the loops are distributed among two sheets. When we consider a selected loop located in the opposite sheet with respect to the antecedent loop, the next loops must fill both sheets of the bilayer structure as additional loops, regardless their specific distribution. Thus, all these loops, except one, take part in the division of the external field flux in exactly the same manner as in the monolayer case, provided that the selected loop is omitted together with the flux passing through this loop. This trick reduces the bilayer system to a monolayer one in the braid-loop topology sense. Thus, we can write the commensurability condition in the bilayer graphene in the case of too short single-loop cyclotron orbits in the following form (as an example, for the subband $n=0,2\uparrow$ of the LLL):

1 $$\begin{array}{rcl}(q-1)\frac{hc}{eB} & = & \frac{S}{N},\\ \nu  & = & \frac{N}{{N}_{0}}=\frac{1}{q-1}=\frac{1}{2},\frac{1}{4},\frac{1}{6},\ldots ,\end{array}$$

where *N* is the number of electrons in each sheet, ${N}_{0}=\frac{BSe}{hc}$ is the degeneracy of any subband, *S* is the surface of the sample (the surface of the single sheet), and *q* is an odd integer (it must be odd to ensure that half of the cyclotron orbit defines the braid^17^, similarly to monolayer case). After omitting a single loop, the next loops must duplicate the former ones, no matter in which way the loops are distributed between both sheets. Thus, only *q* − 1 loops take part in the enhancement of the effective *q*-loop cyclotron orbit size in bilayer graphene, taking into account the same instances of commensurability as in the monolayer case.

It must be emphasized that for multi-loop orbits in bilayer graphene, the total number of loops is still *q* (despite avoiding one loop in the commensurability condition (1)). Therefore, the generators of the corresponding cyclotron subgroup are of the form ${\sigma }_{j}^{q}$, which results in the standard Laughlin correlations with the Jastrow polynomial with exponent *q*. Due to commensurability (1), the resulting main line of filling fractions is $\nu =\frac{1}{q-1}$ (*p*-odd) in the first particle-type subband of the LLL, i.e., in the subband $n\mathrm{=0},2\uparrow$. The even denominators in this main series for the FQHE hierarchy for bilayer graphene coincide well with the experimental observations^4^.

For holes in this subband (holes corresponding to empty states in the almost-filled subband of particle type), we can write $\nu =1-\frac{1}{q-1}$. The generalization to the full hierarchy of FQHE in this subband thus attains the form $\nu =\frac{l}{l(q-2)\pm 1},\quad \nu =1-\frac{l}{l(q-2)\pm 1}$, where *l* > 1 describes the *l*th-order next-nearest neighbours commensurate with the last loop of the *q* − 1 loops, similarly to the monolayer case (as previously, the limit $l\to \infty$ defines the hierarchy for the Hall metal). For the commensurability of the first *q* − 2 loops with *x*th-order (*x* > 1) next-nearest neighbours, $\nu =\frac{xl}{l(q-2)\pm x}$, but similarly to the monolayer graphene, this hierarchy line also has not yet been observed in the LLL of bilayer graphene.

In the following subbands of the LLL, $n=0,2\downarrow$ (assuming that this subband succeeds the former one), the hierarchy is repeated in the same form, but uniformly shifted ahead by 1 (because the commensurability conditions are similar for all subbands with the same *n* due to the same size of the cyclotron orbits). A novelty occurs, however, in the next two subbands of the LLL, $n=1,2\uparrow$ and $n=1,2\downarrow$. Because of the larger size of cyclotron orbits for *n* = 1, the FQHE main series in the first of these subbands of the LLL, $n=1,2\uparrow$, attains the form

2 $$\begin{array}{rcl}\frac{3hc}{eB} & = & \frac{3S}{{N}_{0}} < \frac{S}{N-2{N}_{0}},\\ (p-\mathrm{1)3}\frac{hc}{eB} & = & (p-\mathrm{1)}\frac{3S}{{N}_{0}}=\frac{S}{N-2{N}_{0}},\\ \nu  & = & \frac{N}{{N}_{0}}=2+\frac{1}{\mathrm{3(}q-\mathrm{1)}}=2+\frac{1}{6},\,2+\frac{1}{12},\,2+\frac{1}{18},\ldots \,.\end{array}$$

The generalization of this main series for holes in the subband and to the full FQHE hierarchy in this subband is formulated as follows: for subband holes, $\nu =3-\frac{1}{3(q-1)}$, and for the full FQHE hierarchy in this subband, $\nu =2+\frac{l}{l3(q-2)\pm 1},\nu =3-\frac{l}{l3(q-2)\pm 1}$, $l=\frac{i}{3},\,i=1,2,3,\ldots$ (the Hall metal hierarchy may be obtained in the limit $l\to \infty$).

In the subband $n=1,2\uparrow$, the cyclotron orbit may be larger than the particle separation (similarly to *n* = 1 in monolayer graphene), which allows single-loop commensurability with next-nearest neighbours. For $\frac{3}{{N}_{0}}=\frac{x}{N-2{N}_{0}}$ for *x* = 1, 2, 3, one obtains the fillings rates $\nu =\frac{7}{3},\frac{8}{3},3$. These rates are related with single-loop correlations, similar to those for IQHE (though the first two correspond to non-integer filling rates) and are referred to as FQHE (single-loop). Similarly, to the monolayer case, one can consider a paired state for *x* = 1.5 in the above formula, which corresponds to the perfect commensurability of cyclotron orbits of electron pairs with the separation of these pairs at the electron filling rate $\nu =\frac{5}{2}$. Fillings of the last subband $n=1,2\downarrow$ in the LLL in bilayer graphene satisfy similar conditions because in all subbands with *n* = 1, the cyclotron orbits have the same size and the FQHE hierarchy is only shifted by 1 from the antecedent subband.

The situation changes significantly, however, in the next LL (the first one beyond the LLL). The cyclotron orbits are determined here by the bare kinetic energy with *n* = 2, which gives the cyclotron orbit size $\frac{5hc}{eB}=\frac{5S}{{N}_{0}}$. These orbits are large; thus, multi-loop orbits may be needed only in regions of low electron density close to the subband edges. In the subband $n=2,1\uparrow$, the main multi-loop series and the related full hierarchy for FQHE (multi-loop) are shifted towards subband edges: $\nu =4+\frac{1}{5(p-1)}$, $\nu =4+\frac{l}{l5(p-2)\pm 1},l=\frac{i}{5},i > 1$ (for subband holes, 5− is substituted for 4+ in both of the above formulae). As before, the limit $l\to \infty$ determines the Hall metal hierarchy. Because the orbit size for *n* = 2 may be larger than the particle separation (especially in the central part of the subband), the commensurability of this orbit with the next-nearest neighbours ought to be taken into account. As in the monolayer graphene subband with *n* = 2, one can expect the presence of four (2*n*) satellite FQHE (single-loop) states, symmetrically located around the central paired state. In the subband *n* = 2, 1, ↑, these satellite states occur at $\nu =\frac{21}{5},\frac{22}{5},\frac{23}{5},\frac{24}{5}$ and the central paired state at $\nu =\frac{9}{2}$. Such states are visible in experiments in conventional 2DEG for subbands with *n* = 2—cf. ref.^25^, whereas in bilayer graphene, the experimental picture is different^5^. This peculiarity is again caused by the specific topology of the double-sheet structure.

**Figure 4:**
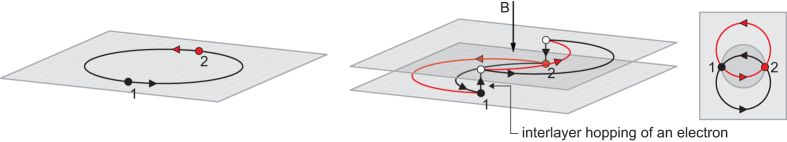
When electrons can hop between two sheets, as in bilayer graphene, the topology of the single-loop interchange of particles may change: both particles can hop between sheets and may be in opposite layers when they traverse their own orbits (centre). In this case, particles may not conserve their mutual distance. This results in leakage of flux of the cyclotron orbit and a smaller-than-nominal orbit (left) that can match particles that lie closer together (right).

**Figure 5:**
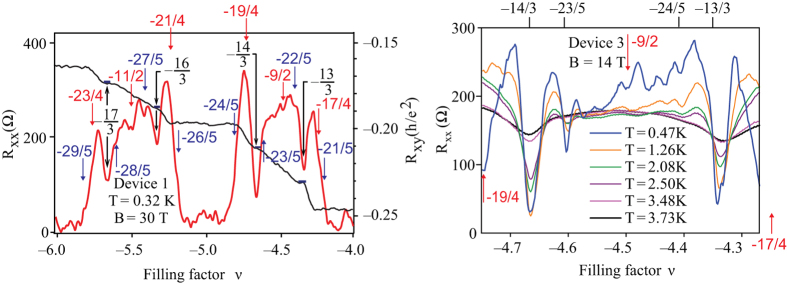
Longitudinal resistivity *R*_*xx*_ measured in bilayer graphene (encapsulated in hBN with open face) for *n* = 2 subbands (first LL)—experiment^5^. The series of fractions with denominator 3 is consistent with the single-loop braid commensurability at the leakage of flux to the opposite sheet in the bilayer structure; the same holds for fractions with denominator 2 or 4. Fractions with denominator 5 correspond to single-loop braid commensurability for *n* = 2 (experiment^5^ is repeated with different samples). [adapted from ref.^5^ under CC-BY 4.0, coloured fractions are added].

**Figure 6:**
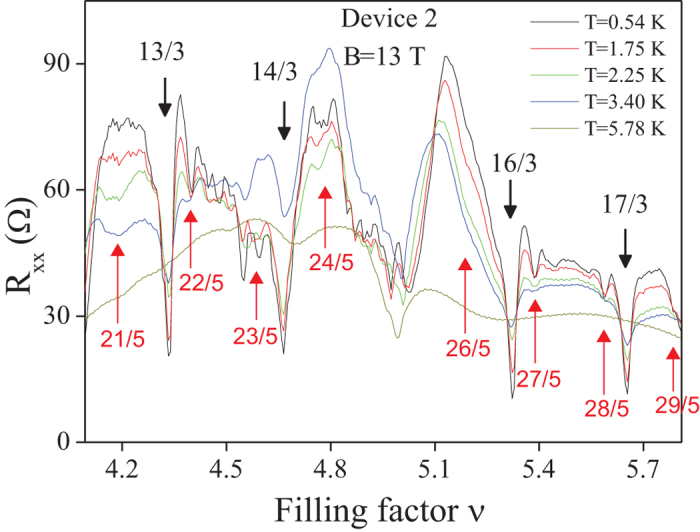
Resistivity *R*_*xx*_ for the bilayer graphene experiment^5^ for the first two subbands with *n* = 2 from the first LL ($\nu \in (4,6)$) (a third sample). The pronounced FQHE features for fractions with denominator 3 for single-loop commensurability (due to leakage of flux between the two sheets of the bilayer structure) and for fractions with denominator 5, also for single-loop braid commensurability, are marked. [adapted from ref.^5^ under CC-BY 4.0, coloured fractions are added].

To solve this puzzle, let us note that in the bilayer system, distinct topological realizations of single-loop orbits may occur that are impossible in the monolayer system. This new opportunity is visualized in Fig. 4, when a part of a single loop is located in one sheet, whereas the rest of this loop in the opposite one, in such a way that particles interchange along cyclotron orbit pieces located in opposite sheets. Such a topology of a single loop can be realized due to interlayer hopping of electrons. Because both electrons may have their individual trajectories in opposite layers when they interchange, the mutual distance between electrons may not be conserved, in contrast to the monolayer case (left panel in Fig. 4). Hence, the braid built from the orbits, as in Fig. 4 (central panel), defines the exchange of electrons that are separated by a distance *smaller* than the orbit size $\frac{5hac}{eB}$ (right panel in Fig. 4). This corresponds to effective reduction of the cyclotron orbit size, or in other words, to a leakage of flux passing through the effective cyclotron orbit. The resulting commensurability can be thus associated with smaller effective cyclotron orbits despite its nominally larger value for *n* = 2. Orbits can change only by integer numbers of flux quanta, thus for the initial nominal flux for *n* = 2, $\frac{5hc}{e}$, one obtains the following final reduced single-loop flux possibilities: $\frac{hc}{e}$, $\frac{2hc}{e}$, $\frac{3hc}{e}$ and $\frac{4hc}{e}$. These effective orbits yield the following new fractions for FQHE (single-loop) due to commensurability with nearest and next-nearest neighbours: $\nu =4+\frac{1}{3}$ and $4+\frac{2}{3}$ for the commensurability of orbit $3\frac{hc}{e}$ with nearest and every second (next-nearest) neighbours, respectively. These fractions are observable experimentally in the first three subbands with *n* = 2 in bilayer graphene^5^—cf. Figs 5 and 6. The corresponding states are more stable because they are associated with single-loop correlations, similarly to IQHE. These states have nothing in common with CFs, as the related correlations are described by single-loop braids. It must be emphasized that the pairs of states $4(5,6,7)+\frac{1}{3}$ and $4(5,6,7)+\frac{2}{3}$ are *not* the particle and hole partners (like the particle $\frac{1}{3}$ and hole $\frac{2}{3}$ multi-loop states in the LLL)—these pairs with denominator 3 in subbands of *n* = 2 LL correspond to single-loop braid commensurabilities of nearest and next-nearest (every second) neighbours, respectively. This is confirmed by the asymmetry in the corresponding local minima of *R*_*xx*_ for these pairs, which is observable experimentally^5^ and illustrates the situation that in states at $4(5,6,7)+\frac{2}{3}$, every second electron is correlated, whereas at $4(5,6,7)+\frac{1}{3}$, all electrons are correlated. Uncorrelated electrons can scatter, which enhances *R*_*xx*_ at $\nu =4(5,6,7)+\frac{2}{3}$. The similar effect of reducing the relative strength of the FQHE features can be associated with the lowering of the fraction of electrons per subband with rising subband number. The gradual lowering of full octets in the first and the second subbands for *n* = 2 and the disappearance of a half-octet of FQHE features in the third subband, together with no features being registered in the fourth subband with *n* = 2 (at least in the temperature range of the experiment, down to 0.5 K)^5^, are consistent with this explanation (the smaller fraction of correlated electrons results in lower energy gain, which can be insufficient to overcome the disorder).

The orbits reduced due to flux leakage to $\frac{hc}{e}$ are too short for single-loop commensurability, whereas orbits $\frac{2hc}{e}$ and $\frac{4hc}{e}$ give $\nu =x+\frac{1}{2}$ and $\nu =x+\frac{1}{4}(\frac{1}{2},\frac{3}{4})$, respectively, *x* = 4, 5, 6, 7. Some traces of these features are noticeable in the experiment, as marked in Fig. 5, which is also consistent with the braid commensurability approach.

### Specific to bilayer graphene FQHE hierarchy change caused by the type of the LLL 2xSU(4) degeneracy lifting

Bilayer graphene has a different subband structure than monolayer graphene and conventional semiconductor 2DEG, as illustrated in Table 1.

**Table 1:** Comparison of subband arrangements in bilayer graphene, monolayer graphene and GaAs 2DEG, and the corresponding filling rate $\nu =\frac{N}{{N}_{0}}$ range (the nominal size of the cyclotron orbit corresponding to *n* is $(2n+1)\frac{hc}{eB}$; however, in the bilayer system, the orbit size can be reduced by flux leakage to the opposite sheet).

Type of system	Subbands of the LLL	Subbands of the first LL	Subbands of the second LL
bilayer graphene	(*n* = 0, 2, ↑, *ν* ∈ (0, 4])	(*n* = 2, 1, ↑, *ν* ∈ (4, 8])	(*n* = 3, 1, ↑), *ν* ∈ (8, 12])
	(*n* = 0, 2, ↓) *conduction band*	(*n* = 2, 1, ↓)	(*n* = 3, 1, ↓)
	(*n* = 1, 2, ↑)	(*n* = 2, 2, ↑)	(*n* = 3, 2, ↑)
	(*n* = 1, 2, ↓)	(*n* = 2, 2, ↓)	(*n* = 3, 2, ↓)
monolayer graphene	(*n* = 0, 2, ↑), *ν* ∈ (0, 2])	(*n* = 1, 1, ↑), *ν* ∈ (2, 6]	(*n* = 2, 1, ↑), *ν* ∈ (6, 10]
	(*n* = 0, 2, ↓) *conduction band*	(*n* = 1, 1, ↓)	(*n* = 2, 1, ↓)
		(*n* = 1, 2, ↑)	(*n* = 2, 2, ↑)
		(*n* = 1, 2, ↓)	(*n* = 2, 2, ↓)
GaAs 2DEG	(*n* = 0, ↑), *ν* ∈ (0, 2]	(*n* = 1, ↑), *ν* ∈ (2, 4]	(*n* = 2, ↑), *ν* ∈ (4, 6]
	(*n* = 0, ↓)	(*n* = 1, ↓)	(*n* = 2, ↓)

**Figure 8:**
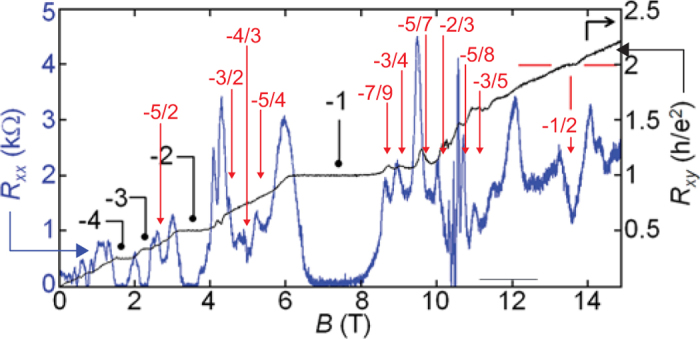
Observation of FQHE at T = 0.25 K in bilayer suspended graphene, magneto-resistance *R*_*xx*_ (blue curve) and *R*_*xy*_ (black curve) at the lateral voltage −27 V, after experiment^4^. Shown in red is the fitting with the cyclotron braid group hierarchy (as in the upper part of Table 2) for mirror valence-band FQHE states, including $\nu =-\frac{1}{2}$ (the mirror fraction to $\nu =\frac{1}{2}$). [adapted with permission from ref.^4^, Copyright 2014 American Chemical Society, coloured fractions are added].

In bilayer graphene, the extra degeneracy of the *n* = 0 and *n* = 1 states results in 8-fold degeneration of the LLL, which is twice the 4-fold spin-valley degeneracy of the LLL in the monolayer case^3^. The Coulomb interaction causes mixing of the *n* = 0 and *n* = 1 states via various schemes induced by stress, deformation, structural imperfections and magnetic field enhancement (as demonstrated in ref.^26^ by exact diagonalization in small models), which lifts their degeneracy in the LLL in bilayer. After the degeneracy lifting, the order of the subbands with *n* = 0 and *n* = 1 occurs to be of particular importance. The sequences *n* = 0, 1 and *n* = 1, 0 lead to different FQHE hierarchies. The FQHE hierarchy for the sequence *n* = 0, 1 is described in paragraph 1.2. However, in the opposite case *n* = 1, 0, when the *n* = 1 subband is filled earlier than the *n* = 0 subband, the hierarchy is different: for the first subband $n=1,2\uparrow$, multi-loop orbits occur for $\nu =\frac{l}{l3(p-2)\pm 1}$ and $\nu =1-\frac{l}{l3(p-2)\pm 1}$, whereas single-loop orbits occur for $\nu =\frac{1}{3},\frac{2}{3}$ and a paired state occurs for $\nu =\frac{1}{2}$. For the next subband (in this ordering), $n=0,2\uparrow$, one obtains the following hierarchy: multi-loop orbits for $\nu =1+\frac{l}{l(p-2)\pm 1}$, $\nu =2-\frac{l}{l(p-2)\pm 1}$ and no single-loop orbits. The origin of such differences is related to the distinct cyclotron orbit sizes for *n* = 1 and *n* = 0. The comparison of reverted orderings of the first two LLL subbands is summarized in Table 2. This evidences that the state at $\nu =\frac{1}{2}$ corresponds to FQHE only when the subband with *n* = 0 is filled earlier than the subband with *n* = 1. In the inverted ordering of these subbands, the state $\frac{1}{2}$ is of the paired type, cf. Table 2. The FQHE hierarchy in bilayer graphene with the visible state at $\nu =-\frac{1}{2}$, observed experimentally in a suspended sample^4^, is shown in Fig. 8.

**Table 2:** Comparison of filling hierarchies in the LLL level in bilayer graphene for two mutually inverted successions of the two lowest subbands: $n=0,2\uparrow$, $n=1,2,\uparrow$ (upper – first two rows) and $n=1,2\uparrow$, $n=0,2\uparrow$ (lower – last two rows). FQHE at $\nu =\frac{1}{2}$ exists for the upper sequence of subbands and it disappears for the lower subband sequence.

LL subb.	FQHE(single-loop), paired–not FQHE, IQHE	FQHE(multi-loop) (*q*–odd, ${\boldsymbol{l}}{\boldsymbol{=}}\frac{{i}}{{\bf{2}}{\boldsymbol{n}}{\boldsymbol{+}}1}$, ${\boldsymbol{i}}{\boldsymbol{=}}{\bf{1}},{\bf{2}},{\bf{3}},{\boldsymbol{\ldots }}$)	Hall metal
(1) $n=0,2\uparrow$	1	$\frac{1}{(q-1)}$ $(\textcolor[rgb]{0,0,0}{\frac{1}{2},\ldots })$, $1-\frac{1}{(q-1)}$, $\frac{l}{l(q-2)\pm 1}$, $1-\frac{l}{l(q-2)\pm 1}$	$\frac{1}{q-2}$, $1-\frac{1}{q-2}$
(2) $n=1,2\uparrow$	$\frac{4}{3}$, $\frac{5}{3}$, $(\frac{3}{2}\,{\rm{paired}})$, 1, 2	$1+\frac{1}{3(q-1)}$, $2-\frac{1}{3(q-1)}$, $1+\frac{l}{3l(q-2)\pm 1}$, $2-\frac{l}{3l(q-2)\pm 1}$	$1+\frac{1}{3(q-2)}$, $2\,-\,\frac{1}{3(q-2)}$
(1) $n=1,2\uparrow$	$\frac{1}{3}$, $\frac{2}{3}$, $(\frac{1}{2}\,{\rm{p}}{\rm{a}}{\rm{i}}{\rm{r}}{\rm{e}}{\rm{d}})$, 1	$\frac{1}{3(q-1)}$ $(\frac{1}{6},\ldots )$, $1-\frac{1}{3(q-1)}$, $\frac{l}{3l(q-2)\pm 1}$, $1-\frac{l}{3l(q-2)\pm 1}$	$\frac{1}{3(q-2)}$, $1-\frac{1}{\mathrm{3(}q-\mathrm{2)}}$
(2) $n=0,2\uparrow$	1, 2	$1+\frac{1}{q-1}$, $1+\frac{l}{l(q-1)\pm 1}$, $2-\frac{1}{q-1}$, $2-\frac{l}{l(q-2)\pm 1}$	$1+\frac{1}{q-2}$, $2\,-\,\frac{1}{q-2}$

One can also consider the situation in the LLL of bilayer graphene when the degeneracy of the *n* = 0, 1 states is lifted in such a way that both levels cross at a certain filling factor *v* ^*^ < 1 (cf. ref.^26^, where mixing between *n* = 0, 1 states has been analysed numerically in small models on the torus or sphere). Let us assume, for an example, that the *n* = 1 subband ($n=1,2\uparrow$) is energetically favourable up to some filling fraction *v* ^*^. At this filling rate, the subband $n=1,2\uparrow$ crosses the subband $n=0,2\uparrow$ and the latter becomes the lower one for $1+{\nu }^{\ast } > \nu  > {\nu }^{\ast }$. The hierarchy of fractional fillings corresponding to such a situation looks like that of ordinary filling of the subband $n=1,2\uparrow$, though with the insertion of the $n=0,2\uparrow$ subband hierarchy. Depending on the value of *v* ^*^, various patterns are possible through a combination of hierarchy patterns, as illustrated in Table 2.

## Comparison with Experiment

More precise observations of FQHE in graphene have been recently obtained through improvements of sampling and measurement techniques, including freely suspended graphene sample measurements^4,11,12^ and graphene samples supported by or encapsulated in hBN layers with similar hexagonal crystal structure^5,9,10^. The range of observation of FQHE reaches the first six subbands in monolayer graphene^10^ and the first eight subbands in bilayer graphene (using the technique of hBN encapsulation with’open face’)^5^.

While the sequence of FQHE fillings in the LLL of monolayer graphene well fits the CF predictions, an explanation of the FQHE filling structure in the next subbands (with *n* = 1) deviates from the CF picture^10–12^. In the bilayer graphene, the incompatibility of the CF model with experimental observations is manifested both in the LLL^4^ and in higher LLs^5^. Various scenarios of breaking of the approximate SU(4) spin-valley symmetry in graphene do not solve this problematic situation despite many theoretical attempts, which evidences the insufficiency of the CF model in this case.

It seems to be more efficient to understand the FQHE in graphene through the braid group-based commensurability approach. The hierarchy for FQHE predicted in this way is consistent with the current experimental data, in both monolayer and bilayer graphene.

**Figure 7:**
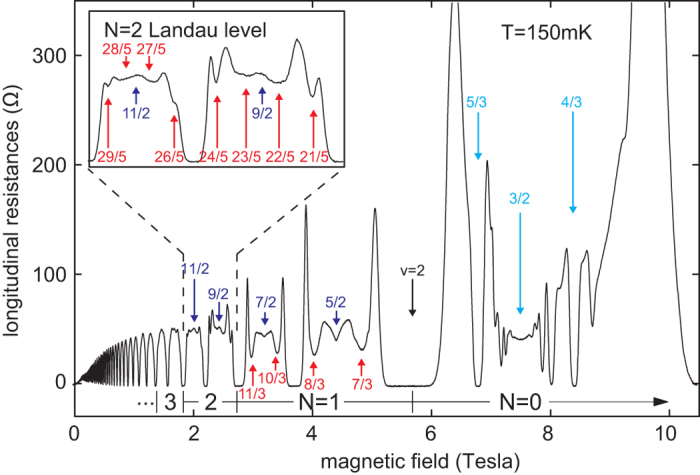
For comparison—measurements of resistivity *R*_*xx*_ in conventional 2DEG for a wide range of magnetic fields corresponding to *n* = 1, 2 in the high-mobility GaAs/AlGaAs heterostructure (following ref.^25^). In red colour, fractions are indicated for the FQHE (single-loop)—doublets with denominator 3 in subbands with *n* = 1 and quartets with denominator 5 for *n* = 2, in accordance with braid commensurability predictions. The pair with denominator 3 for *n* = 0 (blue, 5/3, 4/3) corresponds to 3-loop orbits. At 11/2, 9/2, 7/2, and 5/2, the braid group approach predicts paired states, but for 3/2 and 1/2, it predicts Hall metal. A similar structure of FQHE is predicted for monolayer graphene, though the data for *n* = 2 in monolayer graphene are not available yet.

**Figure 9:**
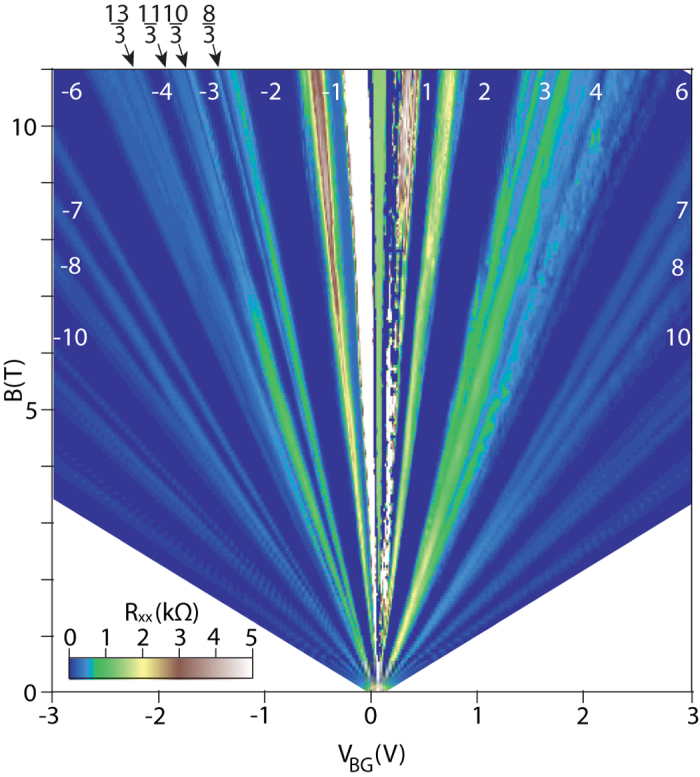
Fan diagram for *R*_*xx*_(*V*. *B*) in monolayer graphene up to 11 T from experiment^10^. The noticeable property is the closeness in value of *R*_*xx*_ of the FQHE features for fractions with denominator 3 for *n* = 1 with those for IQHE, which supports the FQHE single-loop braid correlations in corresponding states, similar to the case of IQHE.

The effectiveness of the CF model in the LLL of graphene monolayer is linked with the fact that exclusively in the LLL, cyclotron orbits are always shorter than the interparticle spacing and additional loops are necessary to exchange neighbouring particles along cyclotron braids. These additional loops can be simulated by auxiliary fictitious field flux quanta attached to CFs (as detailed in SI). However, in the case when the more complicated commensurability conditions support particular FQHE states even in the LLL (known as out-of-CF hierarchy, e.g., $\nu =\frac{5}{13},\frac{4}{11},\frac{3}{10},\ldots$) or in higher LLs, when loops correspond to exchanges of next-nearest electrons, the CF model is insufficient (as proven in SI, Appendices A and B). Simultaneously, the braid commensurability approach reproduces all features described correctly by the CF model and, moreover, explains details that are inaccessible using the CF approach. A generalization of the CF model is especially required in higher LLs because in these levels, the central regions of all subbands with *n* ≥ 1 correspond to cyclotron braid orbits larger than the particle separation, and the multi-loop braids equivalent to CF picture are useless. In this case, the single-loop braid commensurability with next-nearest neighbours is involved beyond the CF concept. In the first LL in monolayer graphene (*n* = 1), the following doublets of fillings are observed: $(\frac{7}{3},\frac{8}{3})$, $(\frac{10}{3},\frac{11}{3})$, $(\frac{13}{3},\frac{14}{3})$, $(\frac{16}{3},\frac{17}{3})$, corresponding to the single-loop braid commensurability of the nearest and next-nearest (every second) neighbours. These doublets are observable in experiments^9–12^. The number of centrally located filling rates for FQHE (single-loop) grows with the LL number as 2*n* (this is observed experimentally in conventional 2DEG: at *n* = 2, four of the fillings with denominator 5 are noticeable, as illustrated in Fig. 7). In monolayer graphene, the repeating doublets of filling ratios (with denominator 3) for *n* = 1 have been observed in very accurate measurements in suspended samples^11,12^, in addition to those on the hBN substrate^9,10^. Worth noting is the observation^10^ that the stability of corresponding FQHE (single-loop) states is of similar strength to that of the IQHE states and is higher in comparison to the FQHE (multi-loop) states, as shown in Fig. 9. This evidences stronger correlations related to single-loop braids, similar to those present in IQHE states.

Note that in higher LLs, multi-loop braids may be needed close to subband edges for small filling rates when the separation of diluted carriers would exceed the orbit size for *n* ≥ 1 (equal to $\frac{(2n+1)BSe}{hc}$). These multi-loop FQHE features are thus shifted towards subband edges and may be washed out by IQHE re-entrant.

In the *n* = 1 LL, new features were observed, in addition to the above mentioned doublets, up to the full octets visible in Fig. 1 in the first two subbands. They are associated with non-vanishing longitudinal resistivity *R*_*xx*_, opposite to the fully developed FQHE states in the LLL. This property is supported by the fact that in agreement with the braid commensurability, not all electrons participate in the corresponding correlated states, but rather every second or every third particle. Scattering of uncorrelated electrons enhances resistivity. These features in the first LL of monolayer graphene (*n* = 1), recently reported^10^ at $\nu =\frac{7}{3},\frac{8}{3},\frac{12}{5},\frac{13}{5},\frac{17}{7},\frac{18}{7},\frac{22}{9},\frac{23}{9},\frac{10}{3},\frac{11}{3},\frac{17}{5},$ $\frac{18}{5},\frac{24}{7},\frac{25}{7},\frac{13}{3},\frac{14}{3},\frac{22}{5},\frac{23}{5}$, are reproduced one to one by the braid commensurability series ν = 2(3, 4) + $\frac{xl}{l3(q-1)\pm 1}$ with $q=3,\,x=2,3,\,l=\frac{i}{3},\,i=1,2,3$, as shown in Fig. 1. One can notice, however, that the FQHE at the filling rates $\frac{7}{3},\frac{8}{3},\frac{10}{3},\frac{11}{3},\frac{13}{3},\frac{14}{3}$ may be single-loop states, which are more stable than the multi-loop ones, which is consistent with the experimental data presented in Fig. 9 and shown in the upper panel of Fig. 1.

In bilayer graphene, the manifestation of FQHE deviates from the CF picture also in the LLL due to the peculiarity of the double-layer topology caused by interlayer hopping of electrons. As was presented in the paragraph 1.2, in the lowest subband of the LLL in bilayer graphene, even-denominator filling fractions for FQHE appear^4^. The commensurability braid group approach for bilayer graphene reproduces all the experimentally observed FQHE hierarchies in the LLL, including the pronounced state at $\nu =-\frac{1}{2}$ —cf. Fig. 8, the illustration in Fig. 2 and Table 2.

Let us emphasize that the FQHE state at $\nu =\frac{1}{2}$ was discovered earlier in the bilayer structure of conventional 2DEG^27,28^, which is also consistent with the commensurability braid group predictions and evidences that this non-CF fraction is caused by the double-layer topology and not by specific material properties of bilayer systems.

Surprisingly, the FQHE state at $\nu =-\frac{1}{2}$ observed in the bilayer structure in the suspended sample disappears, however, in bilayer graphene on hBN substrate^4–8^. We propose to explain this effect by the commensurability braid group approach, noting that the occurrence of the $\pm \frac{1}{2}$ FQHE state depends on the order of the LLL subband degeneracy lifting, as shown in Table 2. We suppose that the external conditions related to the presence of the hBN substrate reverse the ordering in the breaking of 2xSU(4) symmetry of the LLL in bilayer graphene, resulting in the disappearance of the FQHE state at $\nu =\pm \frac{1}{2}$ (as illustrated in Table 2), in comparison to the suspended sample.

The most spectacular observations of FQHE in bilayer graphene were reported recently^5^ for the first LL beyond the LLL, i.e., for *n* = 2 in bilayer graphene (in the first three subbands for filling rate $\nu \in (4,7]$, which is the record for the range for FQHE observations). The unprecedented accuracy of Hall measurement in bilayer graphene encapsulated in hBN for samples with ‘open face’^5^ revealed pronounced FQHE features in subbands with *n* = 2 at filling rates with denominator 3. The fractions with denominator 5 are also noticeable but are weaker in comparison to those with denominator 3 (actually, 2/5 and 3/5 are clearly visible, whereas 1/5 and 4/5 could be identified only as small local bends in the longitudinal resistivity curves). We have explained these astonishing unexpected features, distinct in comparison to conventional 2DEG in *n* = 2 LLs^25^, by a specific double-layer topology of bilayer graphene and by an effective leakage of flux due to electron interlayer hopping, as described in paragraph 1.2. The resulting commensurability hierarchy for FQHE in the first LL with *n* = 2 in the bilayer system (as derived in paragraph 1.2) is perfectly consistent with the experimental data^5^. We have obtained agreement with topological predictions not only for fractions with denominators 3 and 5 but also with denominators 2 and 4 (noticeable at temperature ca. 0.5 K—cf. Fig. 5). The latter features with even denominators are related to the leakage of two flux quanta from the nominal cyclotron orbit at *n* = 2 due to trajectory interlayer hopping, as described in paragraph 1.2. The related correlations for all these features (including fractions with denominator 3 for *n* = 2) are not of CF type, because all correspond to single-loop commensurability instances (as is presented in SI in more detail).

An interesting opportunity to verify the exceptional bilayer FQHE filling structure due to interlayer hopping of braid trajectories arose from a new architecture in Hall measurement, in which additional vertical voltage is applied to the basal bilayer plane. By varying this voltage, the interlayer hopping of electrons in bilayer graphene can be tuned. The applied voltage can open a band gap at the charge-neutrality point and may change the topology of multi-loop trajectories in the bilayer case, reducing them to instances available in the monolayer case. Such an experiment has been performed^7^ for bilayer graphene that is fully encapsulated between two hBN layers, with perpendicular electric field applied by additional bottom and upper electrodes (the displacement field was applied in the range $D\in (-100,100)$ mV/nm). The experiment demonstrates a significant rearrangement of the FQHE hierarchy in the *n* = 0 and *n* = 1 LLL subbands, as expected due to the blocking of interlayer trajectory hopping. The details of this experiment are presented with a discussion in SI. The authors of ref.^7^ argue that the reason for the observed phase transitions is linked with the different ordering of the LLL valley subbands induced by the voltage, since the observed transitions concern not only fractional states but also *v* = 1, 2 accompanying $\nu =\frac{2}{3},\frac{5}{3}$, respectively. A contribution of the change of bilayer topology is, however, also noticeable by inspection of the data presented in ref.^7^ at $\nu =\frac{1}{2},\frac{3}{5},\frac{1}{3}$, as indicated in the experimental curve for *σ*_*xx*_ shown in SI in Fig. 6. The experiment^7^ did not reach the *n* = 2 subbands of the first LL, but in these subbands the expected phase transition due to the reduction of interlayer hopping is predicted to be more explicit and decisive, because pronounced features at filling rates with denominator 3 in subbands with *n* = 2 are generated by interlayer tunnelling (as described in paragraph 1.2) and they should be completely washed out by the blocking of interlayer hopping, in favour of monolayer-type features with denominator 5 (for *n* = 2).

The commensurability braid group approach can also be used to explain the experimental observation of the reduction of the FQHE inter-subband relative strength with growing subband number, and of the intra-subband relative strength, when one compares the so-called electron-hole pairs. Both effects correspond to the lowering of the fraction of electrons participating in correlations. This fraction of electrons is further reduced when there are correlated next-nearest electrons, as occurs for ostensible hole partners, e.g., at $x+\frac{2}{3}$ in the pair with $x+\frac{1}{3}$ (*x*–subband number). For *n* > 1, these hole partners’ are not actually hole partners, but rather electron single-loop states with every second electron braided. The uncorrelated electrons in these states can scatter and enhance *R*_*xx*_, in contrast to dual states with single-loop correlation of all electrons. The gradual diminishing of FQHE strength is visible in all subbands with *n* = 1 of the monolayer graphene^10^, and in all subbands with *n* = 2 of the graphene bilayer^5^. The FQHE energy gain (the activation energy) due to the lowering of the fraction of correlated electrons decreases and eventually drops below the disorder; then the FQHE features disappear in the experiment, as occurred in the fourth subbands of the *n* = 1 and *n* = 2 LLs in the monolayer and the bilayer graphene, respectively^5,10^, at least down to the lowest temperature range in the experimental setup. In both experiments^5,10^, the lowest temperature was 0.5 K. It is quite probable, however, that these very delicate FQHE features might be exposed at mK temperatures. Note also that some FQHE features predicted by the theory may be washed out in experiments by the IQHE re-entrance effect if they are too close to integer fillings (this may concern the poorly visible states at $x+\frac{1}{5}$ and $x+\frac{4}{5}$, where *x* is the subband number in *n* = 2 LL subbands in bilayer graphene).

## Conclusion

We have explained the FQHE hierarchy in graphene in accordance with the recent experimental observations of correlated states, up to the sixth subband in monolayer graphene and up to the eighth subband in its bilayer, revealing deviations from hierarchy schemes known from conventional GaAs 2DEG and apparently going beyond the standard CF model, for subbands with *n* = 1 in monolayer graphene, and in the LLL and the first subband (with *n* = 2) in the bilayer system.

The commensurability of cyclotron braids with interparticle spacing in homogeneous 2D charged systems in a magnetic field is utilized to verify the possibility of arrangement of correlated quantum multiparticle Hall states and to decipher the hierarchy of filling rates for FQHE. By identifying specific topology instances for braids in bilayer graphene caused by the interlayer tunnelling of electrons, we have successfully explained the recent experimental observations of FQHE in the bilayer system. The peculiarity of the FQHE hierarchy, evidenced experimentally in the bilayer graphene in comparison to monolayer graphene and conventional 2DEG, has been clarified in the first eight subbands of the bilayer LL structure.

In the monolayer graphene, it has been demonstrated that in subbands with *n* = 1 there can be present correlated states with single-loop braids that are not equivalent to CFs. The predictions of the braid group commensurability approach reproduce one to one the experimentally observed features in these subbands (*n* = 1). Simultaneously, the relative strengths of the features, mutually compared intra- and inter-subbands, agree with the theory.

A similar consistency between the commensurability braid group theory and the experimental observations holds also in the case of the bilayer graphene. The new opportunities for braid group commensurability in the LLL and in higher LLs were identified in the double-sheet system and attributed to the interlayer hopping of electrons, which leads to a different topology than in the monolayer case and in the ordinary FQHE. The even-denominator main line of the fractional filling hierarchy in the LLL of bilayer graphene is derived in this way, in accordance with experimental observations. The unconventional hierarchy of FQHE that was observed recently in *n* = 2 spin-valley subbands in bilayer graphene is also explained by the same topological arguments, specific to this material. An experimentally observed peculiarity of the FQHE hierarchy in the subbands of the first LL in bilayer graphene with *n* = 2 (different than in monolayer systems for *n* = 2 subbands) has been successfully explained in terms of the specific bilayer system topology caused by the interlayer hopping of electrons. By tuning or even blocking the interlayer tunnelling of electrons by application of voltage perpendicularly to the basal plane of graphene bilayer, the predicted phase transition in the FQHE hierarchy has been experimentally demonstrated, which positively evaluated the topological braid group commensurability approach.

The applied braid group commensurability approach to monolayer and bilayer graphene generalizes the conventional CF model. It is confirmed by up-to-date available experimental FQHE observations in graphene on BN substrate, as well as in suspended samples including monolayer graphene up to the sixth spin-valley subband and bilayer graphene up to the eighth spin-valley subband.

## Electronic supplementary material

Supplementary Information
