# Supplementary material for: Unconventional fractional quantum Hall effect in bilayer graphene
Source: Sci Rep. 2017 Aug 18;7:8720. doi: 10.1038/s41598-017-09166-5 (PMC5562899; doi:10.1038/s41598-017-09166-5)
Supplement: Supplementary file 1 — Supplementary Information [file 41598_2017_9166_MOESM1_ESM.pdf]

SUPPLEMENTARY INFORMATION

(method, supplementary discussion and examples)

Unconventional fractional quantum Hall effect in bilayer graphene

Janusz Edward Jacak[1,*]

[1]Department of Quantum Technologies, Faculty of Fundamental Problems of Technology, Wrocław University of Science and Technology, Wyb. Wyspiańskiego 27, 50-370 Wrocław, Poland
[*]janusz.jacak@pwr.edu.pl

1 The method description

1.1 Composite fermion model of FQHE in topological terms

The correlated Hall states are attributed to the interaction of electrons and may be identified numerically by exact diagonalization of the Coulomb interaction in small model systems. Taking into account the single-particle LL wave functions for the band structure of a particular material (like in graphene modelled with the standard tight binding approximation[1,2]), one can find fractions corresponding to correlated states by numerical minimization of the Coulomb energy in the basis of these functions (as, e.g., for bilayer graphene, presented in Ref. 3). To clarify the physical character of states which exhibit energy minimization, various phenomenological trial wave functions are proposed with different schemes and ideas about the nature of the correlations, including composite fermions (CFs), paired states of Pfaffian type, charge-density waves, spin-correlated states, or Halperin multicomponent generalization of the Laughlin function. The agreement with the exact diagonalization in the LLL of conventional 2DEG supports the CF model[4]. Many fractions in the LLL that are experimentally observed as FQHE are, however, out of reach for the standard CF model (e.g., $\frac{3}{8},\frac{3}{10},\frac{4}{11},\frac{5}{13},\frac{5}{17},\frac{6}{17},\frac{4}{13},\frac{7}{11},\dots$)[5,6].

On the other hand, a different approach to trial wave functions for FQHE has been proposed by Halperin[7] in the form of multicomponent generalization of the Laughlin wave function. Some multicomponent trial functions occur to be very close to the ground states obtained by exact diagonalization. The closeness in energy of various candidates to true ground states, obtained using different approaches with distinct forms of trial wave functions, evidences a convergence to a common nature of FQHE correlations.

To gain insight into this situation, topological arguments can be helpful and they can shed light on both the CF and multicomponent Laughlin-Halperin trial wave functions and their closeness. The topological approach of braid group commensurability allows for tractable discrimination of filling rates and for systematic definition of trial wave functions for FQHE states using the unitary representations of subgroups of the braid group that organize incompressible correlated states at particular filling rates (as detailed in SI Appendices A and B, where the activation energies for these states have been successfully compared with the exact diagonalization energies). The method and resulting trial wave functions simultaneously elucidate the CF construction, revealing its usability limits, and show the linkage to the multicomponent Halperin approach.

The braid group commensurability approach utilizes the Feynman path-integral quantization formalism for multiparticle systems[8–10]. Within this method, the trajectories of interchanging particles (braids) in the configuration space of the N-particle system are considered, including quantum indistinguishability of identical particles. These braid loops (mixing the initial and final enumerations of particles, which are, however, unified due to their indistinguishability) can be attached at any point to the open multiparticle trajectory connecting point $z_1,\dots,z_N$ (the initial point at time instant t) with point $z'_1,\dots,z'_N$ (the final point at time instant t') in the configuration space. Because braid loops are mutually nonhomotopic, the resulting open trajectories with attached distinct braids are also topologically inequivalent (one cannot be transformed into the other in a continuous manner—as illustrated in Fig. 1). Thus, all trajectories fall into disjoint classes of nonhomotopic trajectories (which cannot be unified by continuous deformation). These classes form the domain of the Feynman path integral[8,10],

$$I(z_1,\dots,z_N,t;z'_1,\dots,z'_N,t')=\sum_{l\in\pi_1(\Omega)}e^{i\alpha_l}\int d\lambda_l e^{iS[\lambda_l(z_1,\dots,z_N,t;z'_1,\dots,z'_N,t')]/\hbar},\qquad(1)$$

where $I(z_1,\dots,z_N,t;z'_1,\dots,z'_N,t')$ is the propagator, i.e., the matrix element of the evolution operator of the total system in position representation, which determines the probability of quantum transition from the point $z_1,\dots,z_N$ at time instant t to another

point in the configuration space, $z'_1,\ldots,z'_N$, at time instant t'; $d\lambda_l$ is the measure in the path space sector enumerated by braid group element $l\in\pi_1(\Omega)$; $\pi_1(\Omega)$ is the first homotopy group of the configuration space Ω (it is just called the braid group); $\Omega=(M^N-\Delta)/S_N$; M is the 2D plane; M^N is the N-fold normal product; Δ is the collection of diagonal points in the normal product (when at least two coordinates z_i coincide), subtracted to ensure the conservation of particle number; the quotient structure by the permutation group S_N accounts for quantum indistinguishability of particles; and $S[\lambda_l(z_1,\ldots,z_N,t;z'_1,\ldots,z'_N,t')]$ is the classical action for the trajectory λ_l joining selected points in the configuration space Ω between time instances t and t' and lying in lth sector of the trajectory space. The whole space of trajectories is decomposed into disjoint sectors, which are enumerated by the discrete braid group element index l. The discontinuous decomposition of the domain of the path integral into disjoint sectors (which are topologically inequivalent) precludes the definition of the path measure $d\lambda$ uniformly on the whole space of paths. Thus, for each sector, the measure $d\lambda_l$ must be defined separately and the contributions of all sectors must be summed with unitary factors $e^{i\alpha_l}$ (unitarity is caused by the causality constraint). It has been proved[10] that these unitary factors establish a one-dimensional unitary representation (1DUR) of the braid group. Distinct unitary weights in the path integral (i.e., distinct 1DURs of the braid group) determine different types of quantum particles corresponding to the classical ones. Braids describe particle exchanges, thus their 1DURs assign quantum statistics. Equivalently, the 1DUR of a particular braid defines a phase shift of the multiparticle wave function $\Psi(z_1,\ldots,z_N)$ when its arguments $z_1,\ldots,z_N$ (classical coordinates of particles on the plane) are mutually exchanged according to this braid (let us emphasize that in 2D, these exchanges are *not* permutations[11]).

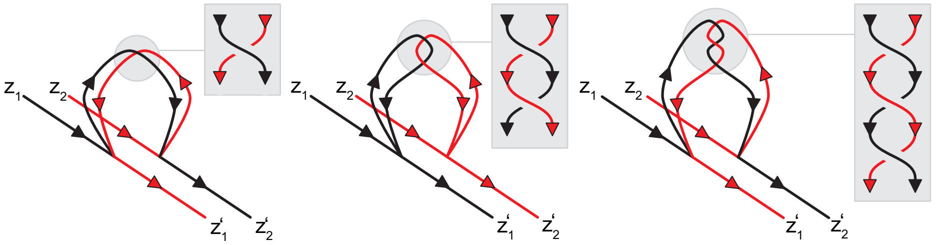

Figure 1. Examples of nonhomotopic trajectories obtained by the addition of various braids to a two-particle trajectory

All quantum multiparticle correlated states (including correlated states of FQHE) must thus be characterized unavoidably by a certain 1DUR of the braid group for a particular system. In 3D, the braid groups of the N-particle systems are always the N-element permutation groups (regardless of charge, interaction or magnetic field presence). There exist only two 1DURs for an arbitrary permutation group, $\sigma_j\to\pm1$ (σ_j, $j=1,\ldots N-1$, are generators of the braid group, i.e., exchanges of jth and $(j+1)$th particles), defining bosons and fermions, whereas in planar systems, the braid groups are different than the permutation group[8,11,12] and their 1DURs are different as well, $\sigma_j\to e^{i\alpha}$, $\alpha\in(-\pi,\pi]$. Various 1DURs define 2D anyons (including 2D fermions for $\alpha=\pi$ and bosons for $\alpha=0$) in the absence of a quantizing magnetic field.

In 2D, for charged repulsing electrons in the presence of a strong perpendicular magnetic field, the braid group approach is not limited to anyons only. A strong magnetic field perpendicular to the basal plane changes significantly the braid group structure, which appears to be the topological factor conditioning the FQHE manifestation, according to the same scheme, even in completely different systems with different single-particle properties, and allows for the CF construction in some specific situations.

Namely, for a strong enough magnetic field, the planar cyclotron orbits may be too short to match neighbouring particles, which are uniformly distributed on the plane (with classical positions fixed by the repulsion of electrons—the classical distribution of 2D charged particles at $T=0$ K is the static triangular Wigner lattice), which precludes the existence of the braid group generators σ_j, i.e., exchanges of neighbouring particles. The braids σ_j, which for charged 2D particles in the presence of a magnetic field must be built from pieces of classical cyclotron orbits, cannot be defined in this case. Too-short σ_j braids, which cannot be implemented, must be rejected from the braid group. Nevertheless, it has been proved[12,13] that the braids remaining in the braid group, which are large enough to match neighbouring particles, form a subgroup of the original group, and this subgroup is called the cyclotron braid subgroup. The generators of the cyclotron subgroups are multi-loop braids—such braids have, in 2D, larger size than single-loop braids[13]. The cyclotron subgroups allow for the definition of quantum statistics in the presence of a strong magnetic field via their 1DURs. In particular, the 1DURs of the cyclotron braid subgroups generated by the fermionic 1DUR of the initial full braid group define CFs and allow for the construction of related multiparticle wave functions for FQHE using symmetry constraints imposed by the form of the cyclotron braid subgroup generators. These wave functions (without the need for any projection onto the LLL, contrary to Jain's idea of CFs) agree with the exact diagonalization on small models, as shown in Appendix B. The trial wave functions do not need to be projected from higher LLs (as in the CF model) but are uniquely defined according to the symmetry imposed by the appropriate 1DUR

and by the particular form of the cyclotron braid group generators—cf. Appendix B for examples.

Construction of appropriate cyclotron braids is possible only at certain specific filling rates of the LLL when the commensurability of cyclotron orbit size with particle separation fixed by the Coulomb interaction is fulfilled. The discrimination of filling rates by this commensurability condition results in a filling hierarchy that is consistent with experimental observations of the FQHE hierarchy.

1.2 FQHE, cyclotron braids and commensurability condition

In the presence of a strong magnetic field, the 2D braids for charged particles must be built from pieces of cyclotron orbits and these orbits of definite size in each LL must precisely fit the interparticle spacing fixed by the Coulomb interaction (in a net of Wigner crystal form). Otherwise, the implementation (definition) of braids is precluded in the presence of a magnetic field in the charged 2D system. Hence, the commensurability of planar braids with particle spacing conditions the definition of the braid group and the determination of the quantum statistics (by 1DURs of the defined braid group). The quantum statistics are a prerequisite to any multiparticle correlated state. Therefore, the commensurability condition determines the magnetic field strength (or equivalently, the Landau level filling rate) at which a correlated state can be formed. Various types of commensurability define all possible filling fractions for correlated multiparticle states in the Hall system.

The archetype of the commensurability in the 2D N-electron system in a strong magnetic field is the accurate fitting of cyclotron orbits of interacting 2D electrons in the LLL to the inter-electron spacing, which occurs at the completely filled LLL, i.e., at filling factor $\nu = \frac{N}{N_0} = 1$, where $N_0 = \frac{B_0 Se}{hc}$ is the LL degeneracy ($\frac{hc}{e}$ is the magnetic-field flux quantum). In other words, the condition $\nu = 1$ is equivalent to the commensurability condition,

$$\frac{S}{N} = \frac{S}{N_0} = \frac{hc}{eB_0}, \tag{2}$$

i.e., the size of the cyclotronic orbit in the LLL at $\nu = 1$, $\frac{hc}{eB_0}$, fits the inter-particle spacing, $\frac{S}{N}$ (S is the size of the system in the 2D plane; in the thermodynamic limit, $\frac{S}{N}$ is constant even if S and N tend to infinity). For $\nu = 1$, the braid group can be thus established and the corresponding correlated state is manifested as the IQHE.

For stronger magnetic fields $B > B_0$, $\nu < 1$, the commensurability (2) fails. This means that ordinary cyclotron orbits, $\frac{hc}{eB}$, are too short and the corresponding braids, σ_j, must be rejected from the braid group as unavailable. However, among the remaining braid group elements, there are still braids σ_j^q, where q is an odd integer, that similarly to σ_j, define exchanges of the jth and $(j+1)$th particles. In contrast to σ_j, the braids σ_j^q realize the exchange with additional $\frac{q-1}{2}$ loops[12,13]. These additional loops 'take away' $\frac{q-1}{2}$ flux quanta when the external field is passing through the planar multi-loop orbit. This is the origin of the auxiliary flux tubes pinned to CFs. In 2D, the external field flux per particle, $\frac{BS}{N}$, is thus reduced by $\frac{q-1}{2}\frac{hc}{e}$, as in the CF concept.

Exclusively in 2D, multi-loop cyclotron orbits share the same external-field flux per particle as the single-loop cyclotron orbit, thus per single loop of a multi-loop orbit must fall only some fraction of $\frac{BS}{N}$–just a flux quantum. Noticeably, this is contrary to the 3D case, in which each wind of the spiral in 3D adds a new surface that is pierced by the same field B, but in 2D not. A flux is the product of a surface and a field, hence its smaller values can be achieved at lower field, conserving the surface. The division of the external $\frac{BS}{N}$ flux into pieces corresponding to each loop is equivalent to the reduction of the effective field for a single loop to the value B_0 at which the flux quantum has the size $\frac{S}{N}$. Hence, in 2D, the multi-loop cyclotron orbits related to σ_j^q have larger size as they are adjusted to the reduced flux portion per loop, and eventually σ_j^q can reach particles that are out of reach for single-loop braids σ_j. The braid cyclotron subgroup generated by σ_j^q, $j = 1, \dots, N-1$, is the proper braid group for $\nu < 1$ provided that the new commensurability condition holds:

$$\frac{S}{N} = \frac{qBSe}{hc}, \tag{3}$$

where the right-hand side of this condition expresses the q-times-larger range of the multi-loop cyclotron orbit in 2D. From this commensurability condition, it follows that $\nu = \frac{N}{N_0} = \frac{1}{q}$, which defines the main line of LLL fillings for FQHE (described by the Laughlin function with the qth-order Jastrow polynomial). The multiparticle wave function of N correlated 2D charged interacting particles (electrons) in a magnetic field must transform according to the 1DUR of the related cyclotron braid subgroup[8,10,14]. This feature, together with the requirement that the multiparticle function in the LLL must be a holomorphic function of $z_1, \dots, z_N$ ($z_j = x_j + iy_j$—complex coordinate on the plane of jth particle), uniquely results in form of the Jastrow polynomial: $\prod_{i>j}^N (z_i - z_j)^q$ (multiplied by a factor that is independent of particle interchanges—in the Laughlin function $e^{-\sum_{i=1}^N |z_i|^2/4l_B^2}$, where l_B is the magnetic length). The latter term is common for all states from the LLL, thus the derivation of the Laughlin function is reduced to the determination of the polynomial factor, which is uniform with respect to all particles and can be deduced according to the 1DUR and the form of a generator of the related cyclotron braid subgroup.

In 2D, the multi-loop cyclotron orbits have larger size, which allows the braids σ_j^q (where q is an odd positive integer) to fit the interparticle separation too large for single-loop exchanges. This opportunity, however, occurs only at some 'magic' fractional fillings of the LLL—the same ones at which FQHE is observed. The surface spanned by a 2D orbit is the same regardless of its multi-loop character, hence, the portion of the flux per loop diminishes for a multi-loop orbit in comparison to a single-loop orbit, which subsequently results in the growth of the multi-loop orbit size, as illustrated in Fig. 3.

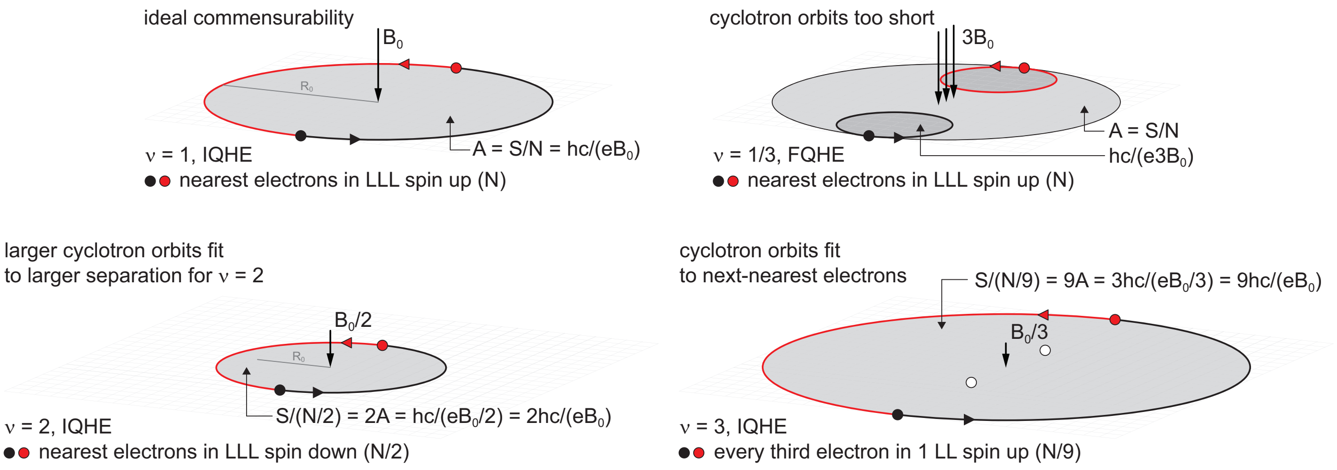

Figure 2. Illustration of the commensurability between the cyclotron orbits (schematically drawn of circular shape) and the interparticle spacing. At $\nu = 1$, nearest particles may exchange positions along perfectly accommodated single-loop cyclotron orbits (a). For stronger fields $\nu < 1$, single-loop cyclotron orbits are too short to match neighbouring particles (b). For $\nu = 2$, orbits again fit the interparticle spacing in the spin-down subband of the LLL (c). For $\nu = 3$, i.e., for complete filling of the spin-up subband of the first LL, cyclotron orbits fit accurately every third particle separation in this subband (d). In cases (a), (c), and (d), we address the IQHE, whereas in (b) at $\nu = \frac{1}{3}$, we address the FQHE due to the larger three-loop orbits.

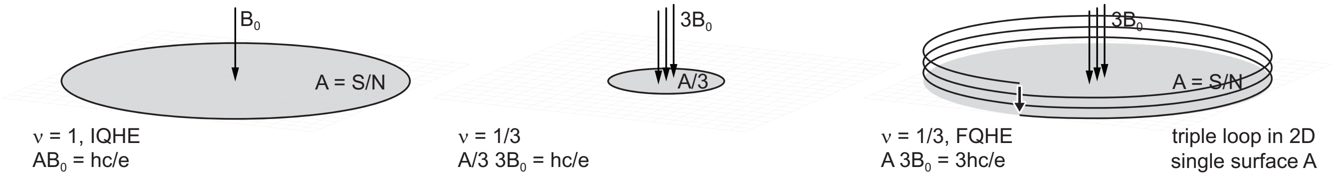

Figure 3. Schematic illustration of cyclotron orbit enhancement in 2D due to the multi-loop trajectory structure (the third dimension is added for visual clarity). The single-loop orbit fits the particle separation at $\nu = 1$ (left). For the multi-loop orbit in 2D, an external magnetic-field flux must be divided among all loops. Each loop is associated with only a fraction of the external-field flux, which leads to an enhancement of the loop size (right).

In Fig. 3 (left), the scheme of the cyclotron orbit at the magnetic field B_0 is shown, as adjusted to the quantum of magnetic field flux, i.e., $B_0A = \frac{hc}{e}$. This is the definition of the single-loop cyclotron orbit size A for B_0 in the LLL. A fits the interparticle separation in the case of the completely filled LLL, $\frac{S}{N}$; S—the sample area, N—the number of particles. If only single-loop orbits are considered, then at larger fields, e.g., $q = 3$–times larger, $3B_0$, the cyclotron orbit that is adjusted to the flux quantum is too small in comparison to the interparticle separation $\frac{S}{N}$—as is sketched in the central panel of Fig. 3. However, in the case when three-loop orbits are considered, exclusively in 2D space, the external flux $\frac{3B_0S}{N} = 3B_0A$ passing through this orbit must be divided among three loops. In the case of uniform division among the loops, B_0A of the total $3B_0A$ flux is associated with each loop. Therefore, in this situation, each loop adjusted to the flux quantum $\frac{hc}{e} = B_0A$ has the size A, which is the same as for the single-loop orbit in a three-times-weaker magnetic field. The three loops contribute together to the total flux $3B_0A$ per particle as needed—cf. Fig. 3 (right). This means that the three-loop orbits surprisingly fit the interparticle separation $\frac{S}{N} = A$.

Because the braid group generator must be defined by half of the cyclotron orbit (cf. Fig. 4), the braid with one additional loop corresponds to the cyclotron orbits with three loops—such a braid generator has the form σ_j^3. The group generated by σ_j^3, $j = 1, \ldots, N-1$, (new elementary braid exchanges) is obviously the subgroup of the original braid group because its generators σ_j^3 are built from original group generators σ_j. This subgroup is called the cyclotron braid subgroup. It is clear

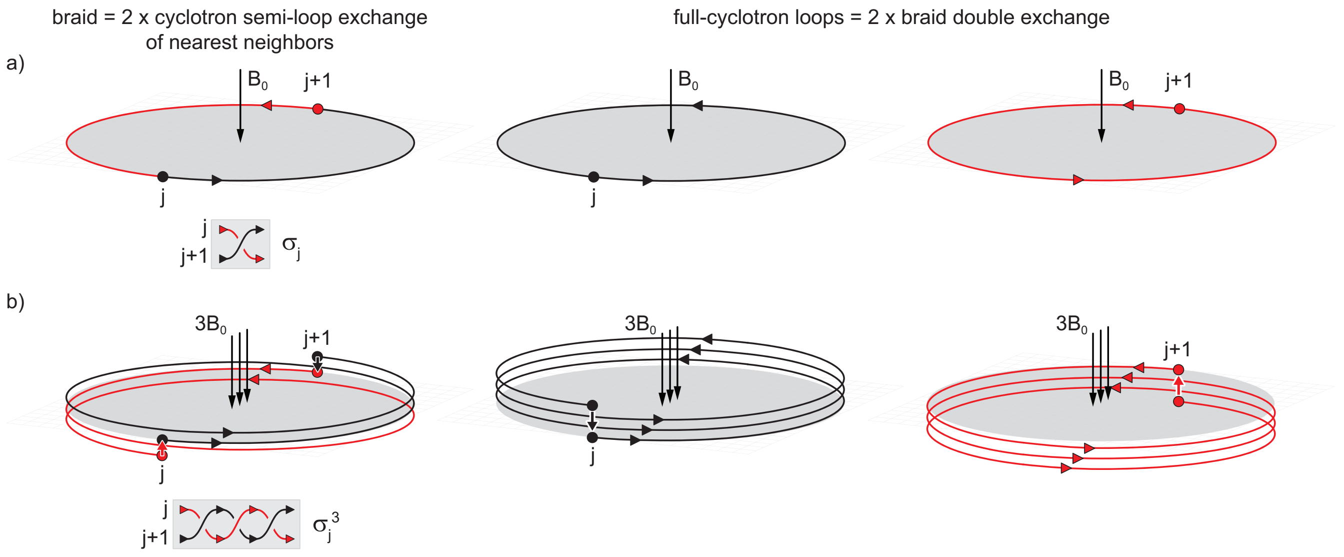

Figure 4. The geometrical presentation of the braid generator σ_j (a)—it corresponds to the ordinary single-loop exchange of neighbouring particles, jth and $(j+1)$th (left). The cyclotron semi-orbits of both particles may realize together this braid at $\nu = 1$. The closed cyclotron full-orbits correspond to the double exchange of matched particles (right). The three-loop braid generator σ_i^3 (b) must be considered at $\nu = \frac{1}{3}$ to match neighbouring particles in a three-times stronger magnetic field $3B_0$. The additional loops are needed in this case to enhance an effective cyclotron radius (the third dimension is added for visual clarity).

that the 1DURs of this subgroup define the statistics of 2D charged particles in sufficiently strong magnetic fields, i.e., in fields that correspond to the fractional fillings of the LLL, provided that the commensurability condition is fulfilled (in the presented example, for $\nu = \frac{1}{3}$). The generalization to more loops that are attached to the braid generator one by one, results in the doubling of the number of loops in the multi-loop cyclotron orbits, and thereby in the filling fractions $\nu = \frac{1}{q}$, where q is an odd integer.

This approach satisfactorily reproduces the hierarchy of the FQHE filling fractions in the LLL in agreement with experimental data (as illustrated in Appendix A). It can be generalized to higher LLs, also in exact agreement with the experimental observations[15]. The generalization to higher LLs involves the observation that the filling fractions corresponding to the commensurability condition can be written as

$$x\frac{S}{N} = \frac{(2n+1)hc}{eB}, \tag{4}$$

where x is a positive integer. The cyclotron orbits in higher LLs are larger than those in the LLL due to higher energy, and in the nth LL are of size $\frac{(2n+1)hc}{eB}$. These larger orbits may fit equidistantly separated particles, though not all but rather every xth particle (next-nearest neighbours of the xth order). Thus, the commensurability (4) also allows for the definition of the generators σ_j' in the form of ordinary single-loop braids linking every xth particles. This occurs in the completely filled higher LLs ($n \geq 1$). The resulting statistics (expressed by the 1DUR of these braid groups with longer braids for $n > 1$) are the same as for IQHE (the longer braids are single loops, as for IQHE at $\nu = 1$). Remarkably, such an opportunity for commensurability may be encountered in higher LLs, not only for completely filled levels but also at some fractional fillings, which has been described in Ref. 15, in satisfactory agreement with the currently available experimental observations up to the third LL for 2DEG in conventional semiconductor Hall systems[16–18] (cf. also Fig. 7 in the main text). It must be emphasized, however, that the situation described above of cyclotron orbits that are too large in comparison to the particle separation (and fitting every xth particle with $x > 1$, i.e., fitting next-nearest neighbours) may occur only for $n \geq 1$, where n is the LL number. Only in the LLs with $n \geq 1$ (i.e., for weaker magnetic field in comparison to the LLL with $n = 0$) are cyclotron orbits of sufficiently large size to match every second particle, every third particle, and so on. This is because the size of the cyclotron orbits in the nth LL grows proportionally to the factor $2n+1$ in the Landau kinetic energy, i.e., the cyclotron orbit size attains the value $\frac{(2n+1)hc}{eB}$ for particles with kinetic energy $(2n+1)\frac{eB}{2mc}$ in the nth LL. Moreover, for $n \geq 1$, cyclotron orbits that are too short may also sometimes occur, but not always, in contrary to the LLL ($n = 0$). In higher LLs, too-short cyclotron orbits may be encountered close to the subbands edges, i.e., for sufficiently small density of particles and thus for separation exceeding the size of the large cyclotron orbits at $n \geq 1$. The quantization of the transverse resistance R_{xy} at filling rate ν (also in higher LLs)

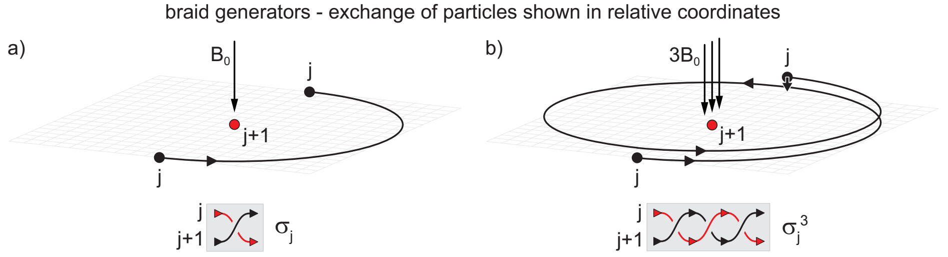

Figure 5. The geometrical presentation of the braid generators σ_j (a) and σ_j^3 (b), visualized in relative coordinates of the jth particle with respect to the $(j+1)$th one. In these coordinates, the latter is at rest in the centre of the braid loop. This braid loop has size equal to the double cyclotron radius, fitted to the interparticle spacing of nearest particles. In relative coordinates, σ_j^3 acquires an additional loop (similar to the cyclotron semi-loops of both particles shown in Fig. 4) (the third dimension is added for visual clarity).

is always similar to that for ordinary FQHE in the LLL, i.e., is equal to $\frac{h}{e^2\nu}$, regardless of whether the correlations expressed by the exponent in the Jastrow polynomial represent single-loop or multi-loop braid exchanges; both are accessible in higher LLs, in contrast to the LLL.

The FQHE phenomena in graphene monolayer and bilayer supply an opportunity to verify the cyclotron braid group commensurability approach because the 'relativistic' LL structure is different in comparison to conventional GaAs 2DEG. The double-sheet topology in bilayer graphene is different than its counterpart in the monolayer case, which causes different commensurability conditions in the bilayer system than in the monolayer one.

2 Supplementary discussion and examples

2.1 FQHE commensurability in graphene

The pseudo-relativistic form of the LL energy emerges from the linearity in momentum of the local Hamiltonian in the vicinities of Dirac points. The massive degeneracy of each LL subband in graphene is, however, the same as in the conventional 2DEG and is equal to $\frac{BS}{hc/e}$ (where B is the external magnetic field, S is the sample surface, and $\frac{hc}{e}$ is the magnetic-field flux quantum). Nevertheless, the number of subbands per LL in graphene is different than in a conventional semiconductor case and equals four in graphene, which corresponds to the Zeeman spin splitting and to the valley pseudo-spin splitting (which is absent in conventional semiconductors) due to the coexistence of two inequivalent Dirac points with two sublattices in the crystal lattice of the graphene sheet[1]. The Zeeman splitting in graphene is small and the valley splitting is small as well[1,19], thus the approximately four-fold spin-valley additional degeneracy may be assumed (referring to SU(4) symmetry). The LLL subbands are divided between particles and holes from the conduction and valence bands[1]. Hence, the bottom of the LLL is shifted upward by 2 in terms of the filling factor (in the monolayer case). The SU(4) symmetry in graphene is broken, and spin-valley subbands of LLs are usually not mixed and are filled with electrons separately. In bilayer graphene, an 'accidental' degeneracy of the $n=0$ and $n=1$ oscillatory states in the LLL[2] additionally enriches the symmetry breaking, which matches experimental observations (as illustrated in paragraph 1.3 of the main text).

The hierarchy of FQHE in the LLL of monolayer graphene is satisfactorily displayed by the CF model. The cyclotron orbit size in the first subband of the LLL, $n=0,2\uparrow$, is equal to $\frac{hc/e}{B}=\frac{S}{N_0}$. This orbit size is smaller than the interparticle spacing expressed by $\frac{S}{N}$ (as $N<N_0$), thus the multi-loop braids with enhanced size are needed to match neighbouring particles[12,13]. The multi-loop orbits in this simplest commensurability instance, $q\frac{S}{N_0}=\frac{S}{N}$, are equivalent to the CF structure (as detailed in Appendices A and B to this SI), which gives $\nu=\frac{N}{N_0}=\frac{1}{q}$ (where q is an odd integer[13]). When the last loop of the multi-loop cyclotron orbit is commensurate with the separation of every lth particle (as in the lth LL), whereas the $q-1$ previous loops take away an integer number of flux quanta (commensurate with the nearest-neighbouring particles), we obtain the hierarchy of fillings for FQHE in this LLL subband in the following form (the same as for the CF model): $\nu=\frac{l}{l(q-1)\pm 1}$, $\nu=1-\frac{l}{l(q-1)\pm 1}$, where $l=1,2,\ldots$ and minus in the denominator corresponds to the possibility of the reverse eight-figure orientation of the last loop with respect to the antecedent loop in the multi-loop orbit.

The limit $l\to\infty$ in the above formulae, $\nu=\frac{1}{q-1}$, $\nu=1-\frac{1}{q-1}$, corresponds to the situation when the residual flux passing through the last loop tends to zero. This means that the last loop can reach, in such a case, the infinitely distant particles, as for fermions at the absence of a magnetic field (Hall metal state).

One can also observe that other variants of commensurability may concern multi-loop orbits. Namely, each loop of the multi-loop structure may in principle accommodate the particle separation in a different and mutually independent manner,

matching nearest or next-nearest neighbours under various schemes. One such possibility may correspond to the situation in the q-loop orbit when $q-1$ loops accommodate every xth particle ($x=1,2,3,\dots$), whereas the last one fits every lth particle separation. This commensurability scheme with $x>1$ is observed in ordinary 2DEG Hall systems (GaAs) in the LLL for the exotic fractions, e.g., $\nu=\frac{4}{11},\frac{5}{13},\frac{3}{8},\frac{3}{10},\dots$ (beyond the conventional CF hierarchy corresponding to $x=1$, as explained in Appendices A and B of this SI). Interestingly, this series of exotic FQHE filling fractions has not yet been observed in the LLL in graphene. Realization of any state is conditioned on the energy competition between various candidates and on the disorder present in the sample at the temperature at which the observation is carried out.

Beyond the LLL, one must take into account that cyclotron orbits are larger than those in the LLL. If n denotes the LL number, then the cyclotron orbit size in the nth LL is equal to $\frac{(2n+1)S}{N_0}$. Only for small number of electrons in subbands with $n\geq 1$ (close to the subband edge) are these single-loop orbits shorter than the electron separation. The related commensurability with q-loop orbits agrees with the CF model (as described in the main text). Nevertheless, due to the larger cyclotron orbits in $n\geq 1$ subbands, a new type of commensurability is possible, but it is not accessible in the LLL and is beyond the CF model. This new commensurability occurs when cyclotron orbits $\frac{(2n+1)S}{N_0}$ fit with the next-nearest electrons. In such a case, the single-loop orbit (large enough in subbands with $n\geq 1$, in contrast to $n=0$) can fit with every xth particle ($x>1$). From this new commensurability opportunity, one finds fractions with denominators $2n+1$ in subbands with $n\geq 1$, which are observable in experiments in higher LLs in the conventional 2DEG[16] (cf. Fig. 7 in the main text) and in the monolayer graphene (as presented in the main text). These states are related to single-loop orbits; thus, they have nothing in common with CFs, even though in the case of $n=1$, the corresponding filling rates have the denominator 3.

Another opportunity for commensurability occurs for orbits commensurate with next-nearest neighbours in higher LLs and corresponds to the nesting of multi-loop orbits with next-nearest electrons. This situation is also out of reach for the CF model, but perfectly agrees with experimental observations (as summarized in Fig. 1 in the main text). Worth noting is the one-to-one consistency of the predicted hierarchy, $\nu=\frac{7}{3},\frac{8}{3},\frac{12}{5},\frac{13}{5},\frac{17}{7},\frac{18}{7},\frac{22}{9},\frac{23}{9},\frac{10}{3},\frac{11}{3},\frac{17}{5},\frac{18}{5},\frac{24}{7},\frac{25}{7},\frac{13}{3},\frac{14}{3},\frac{22}{5},\frac{23}{5}$, with experimental observations in the first three subbands of the $n=1$ LL in the monolayer graphene at ultra-low temperatures[20].

2.2 Inter-layer hopping of trajectories in bilayer graphene

In bilayer graphene, the topology of the braid trajectories is different than in the monolayer system. The bilayer graphene behaves as a planar Hall system with the massive degeneracy of each LL subband the same as in the monolayer case, $N_0=\frac{BSe}{hc}$ (where S is the surface of a single sheet). Nevertheless, the bilayer system is not strictly two dimensional and trajectories may be distributed among both sheets, each with its own surface. This makes a difference in the comparison with the monolayer case because each sheet contributes to the total flux of the external magnetic field independently, which essentially affects the cyclotron orbit size and braid commensurability condition, in excellent consistency with experimental observations. The sharing of orbit loops between two sheets may occur both for multi-loop orbits and for single-loop ones. In the latter case, a single loop can partially hop between layers (as illustrated in Fig. 5 of the main text). The inter-layer hopping of trajectories can be handled using the braid group approach, but cannot be accounted for in the framework of the CF model. Therefore, the CF model does not agree with experimental observations of the FQHE hierarchy in the bilayer graphene[21–23].

The multi-loop orbits can be distributed between two layers of bilayer graphene due to interlayer hopping of electrons. However, via the avoidance of a single loop (taking away the flux passing through this selected loop in one sheet of the bilayer), the commensurability condition for the bilayer system eventually repeats the nesting of loops as in the monolayer case. The simplest such instance is encountered in the LLL (subband $n=0,2\uparrow$) with a three-loop cyclotron orbit located in both sheets of the bilayer graphene (as illustrated in Fig. 2 in the main text), which leads to the filling fraction $\nu=\frac{1}{2}$ for FQHE[21]. This state cannot be explained by the CF model. The CF model, similarly to the braid group model in the monolayer case, predicts a Hall metal state at $\nu=\pm\frac{1}{2}$.

The consequences of the interlayer trajectory hopping in the bilayer graphene are described in the main text in accordance with the available experimental observations.

The oddness of the bilayer topology is manifested sharply in the LL with $n=2$, in which the nominal cyclotron orbit size is equal to $\frac{5hc}{eB}=\frac{5S}{N_0}$. As explained in the main text (paragraph 1.2), these large orbits may be nested to accommodate smaller particle separation than that which follows from the nominal orbit size. Because single loops can also hop between sheets of graphene bilayer, they may be not symmetric as in the monolayer case (this is illustrated in Fig. 4 in the main text). Such an opportunity occurs just for $n\geq 2$ and explains the series of experimental observations of the FQHE hierarchy in the graphene bilayer for the first LL (with $n=2$). These interlayer hopping orbits give the following new fractions for FQHE (single-loop) due to the commensurability with nearest and next-nearest neighbours: $\nu=4(5,6,7)+\frac{1}{3}$ and $4(5,6,7)+\frac{2}{3}$ (see the main text, paragraph 1.2). These pairs of states with denominator 3 in subbands of $n=2$ LL are *not* the particle and hole partners (in contrast to particle $\frac{1}{3}$ and hole $\frac{2}{3}$ multi-loop CF-type states in the LLL)—these pairs correspond to single-loop braid commensurabilities of nearest and next-nearest (every second) neighbours, respectively. The small experimentally observed asymmetry in the corresponding local minima of R_{xx} for these pairs is thus consistent with the difference in the

correlations—for $4(5,6)+\frac{2}{3}$, every second electron is correlated, whereas for $4(5,6)+\frac{1}{3}$, all electrons are correlated. If only the next-nearest neighbours are correlated, the remaining uncorrelated electrons may scatter, enhancing the resistance. The same phenomenon is observed in the LLL of conventional (GaAs) 2DEG, where the local minima of R_{xx} are nonzero if the related states are described by correlations of next-nearest neighbours (the filling rates, which are out of the CF series), as indicated in Fig. 8 (right) in the SI.

Orbits effectively reduced by inter-layer hopping (as described in the main text in paragraph 1.2) to $\frac{hc}{e}$ are too short for single-loop commensurability, whereas orbits $\frac{2hc}{e}$ and $\frac{4hc}{e}$ give $\nu=\frac{1}{2}$ and $\nu=\frac{1}{4},\frac{1}{2},\frac{3}{4}$, respectively. These features are also noticeable in experiments, in R_{xx} plots—cf. Figs. 5 and 6 in the main text.

The convincing test of the presented topological braid group approach to the bilayer system is the experimental evidence of the state $\nu=-\frac{1}{2}$ in the suspended graphene bilayer[21]. The explanation of the disappearance of this feature in the bilayer graphene sample deposited on the hBN substrate[22,24] also agrees with the topological model. The latter can be addressed by the different scheme for the breaking of the doubled SU(4) symmetry in the LLL in bilayer graphene when it is influenced by the hBN substrate. Structural factors such as stress, deformation and crystalline imperfections cause lifting of the valley degeneracy. The Coulomb interaction causes mixing of the $n=0$ and $n=1$ states in the bilayer, which lifts their 'extra' degeneracy within the LLL in the bilayer graphene. We have shown that two possible types of symmetry breaking, resulting in different orders of states, $n=0,1$ or $n=1,0$, lead to distinct FQHE filling hierarchies, as detailed in the main text in paragraph 1.3. The state at $\nu=\frac{1}{2}$ ($\nu=-\frac{1}{2}$) corresponds to FQHE only when the subband with $n=0$ is filled earlier (has lower energy) than the subband with $n=1$ (cf. Tab. 3 in the main text).

Effectiveness of the CF model in the LLL in the monolayer system is linked with the fact that exclusively in the LLL, cyclotron orbits are always shorter than the interparticle spacing and additional loops are necessary to exchange neighbouring particles along braids. These additional loops can be simulated by auxiliary fictitious field flux quanta attached to CFs. However, in the case when the more complicated commensurability conditions support particular FQHE states in the LLL (e.g., for $\nu=\frac{5}{13},\frac{4}{11},\frac{3}{10},\dots$) or in higher LLs, then the CF model is insufficient, cf. Appendix A of this SI). The braid group approach reproduces all features described correctly by the CF model and, moreover, explains hierarchy details that are inaccessible to the CF approach. The usefulness of the CF model is particularly limited in higher LLs because in these levels, the CF-equivalent multi-loop commensurability is needed only close to the subband edges, whereas the central regions of all subbands in higher LLs correspond to cyclotron orbits that are larger than the particle separation.

The braid group approach predicts doublets of FQHE states at $n=1$ LLs. These doublets in monolayer graphene are observable in experiments[20,25–27]. The number of centrally located filling rates for FQHE (single-loop) grows with the LL number as $2n$. Four fillings with denominator 5[16] are observed experimentally in conventional 2DEG at $n=2$, as illustrated in Fig. 7 in the main text. Worth noting is the observation[20] that stability of the corresponding FQHE (single-loop) states is of similar strength to those of IQHE states and higher in comparison to FQHE (multi-loop) states, as shown in Fig. 9 in the main text. This indicates that stronger correlations related to single-loop braids at fractional fillings in higher LLs, similar to those in IQHE states, are present.

2.3 Additional confirmation in the experiment

In bilayer graphene, the manifestation of FQHE deviates from the CF picture, also in the LLL due to the peculiarity of the double-layer topology. As was presented in paragraph 1.2 of the main text, in the lowest subband of the LLL in bilayer graphene, the even-denominator filling fractions for FQHE appear[21] (Figs. 8 and 9 and Tab. 2 in the main text).

Note that the FQHE state at $\nu=\frac{1}{2}$ has been discovered earlier in the bilayer structure of conventional 2DEG[28,29], which evidences that this fraction is caused by the double-layer topology and not by specific material properties or a band structure of the particular bilayer system.

The controversial observations of FQHE in bilayer graphene were also reported[30] in the first LL beyond the LLL, i.e., for $n=2$ in bilayer graphene (in the first three subbands with $n=2$, i.e., for $\nu\in(4,7]$), revealing pronounced FQHE features in the subbands with $n=2$ at filling rates with denominator 3 (which is characteristic for $n=1$ in the monolayer case). Nevertheless, the commensurability hierarchy for FQHE in the LL with $n=2$ in the bilayer system derived in the main text, including an interlayer-hopping trajectory in bilayer graphene, appears to be perfectly consistent with the experimental data[30]. It has been demonstrated that the fractions with denominator 3 in LL subbands with $n=2$ are not of CF type, but rather corresponding to single-loop braid correlations. We have observed agreement with the relevant topological predictions not only for fractions with denominators 3 and 5 but also with denominators 2 and 4 (some experimental evidence for these states in the R_{xx} at low temperature, ~ 0.5 K, can be identified, as indicated in Fig. 6 in the main text). The related correlations for all these features (including fractions with denominators 3, 5, 4, and 2 for $n=2$) are not of CF type, because they all correspond to single-loop commensurability instances.

In view of the topological effects in bilayer graphene that result from the hopping of electrons between graphene sheets and cause a specific cyclotron braid commensurability, one can expect the phase transition to be observed in the FQHE hierarchy

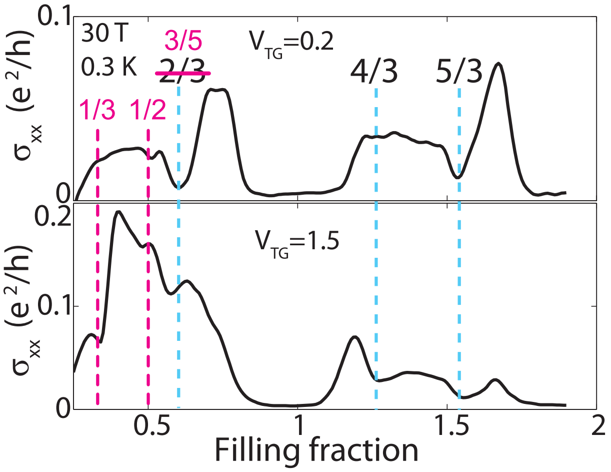

Figure 6. Experimental evidence (cf. Ref. 23) for a phase transition in bilayer graphene due to the reduction of the inter-layer hopping by control of the vertical voltage applied to the basal plane—the change of the development of the 1/2 and 3/5 states (the latter by mistake marked in the figure in Ref. 23 as 2/3) is noticeable (they are visible at low voltage), along with depression of the 1/3 state (it is visible at higher voltage). This agrees with the structure of fillings in bilayer graphene shown in Fig. 8 of the main text and with predictions of the braid group approach.

and induced by tuning of the interlayer hopping of electrons. The hopping of electrons can be tuned by the vertical electric field applied perpendicularly to the basal plane. The applied vertical voltage can open a band gap at the charge-neutrality point and may change the topology of multi-loop or single-loop trajectories in the bilayer case, reducing them to only the instances available in the monolayer case. Such an experiment has been performed[23] for fully encapsulated bilayer graphene between two hBN layers with perpendicular electric field applied (the displacement field $D \in (-100, 100)$ mV/nm). This experiment demonstrates a significant rearrangement of the FQHE hierarchy in $n = 0$ and $n = 1$ LLL subbands of the bilayer sample. The authors of Ref. 23 argue that the reason for the observed phase transitions is linked with a different ordering of LLL valley subbands, which is induced by the voltage when the observed transitions correspond to fractional states $\nu = \frac{2}{3}, \frac{5}{3}$, i.e., in the first two subbands of the LLL. A closer inspection of the data presented in Ref. 23 reveals, however, the transition at $\nu = \frac{1}{2}, \frac{3}{5}$, as shown in Fig. 6, which agrees with blocking of the inter-layer hopping quenching states at $\frac{1}{2}$ and $\frac{3}{5}$ (typical for a bilayer, cf. Fig. 8 in the main text) in favour of the CF state $\frac{1}{3}$ (of CF type, as in the monolayer). The experiment[23] did not reach the $n = 2$ subbands of the first LL, but in these subbands the expected phase transition due to the reduction of interlayer hopping might be even more explicit. One can expect that by blocking the interlayer hopping, the pronounced features with denominator 3 in the $n = 2$ subbands (typical for the bilayer graphene and induced by the interlayer hopping of trajectories, as detailed in the main text in paragraph 1.2) should be cancelled in favour of the fractions with denominator 5 present in the monolayer systems for the $n = 2$ LL subbands. Unfortunately, such an experiment is limited by the applied technique, called 'open face' hBN encapsulation, which allows for observation of $n = 2$ LL subbands[30] but generates a problem with the location of the top electrode for the vertical voltage[23]. Overcoming of this technical obstacle might provide, however, additional and decisive evidence for the role of the interlayer hopping of orbits in shaping the FQHE hierarchy. It would be a definitive test for verification that experimentally observed pairs of states in higher LLs in bilayer as well as monolayer Hall systems are not of CF type, despite the characteristic denominator 3. In this respect, one can also note that in the experiment[30], the doublets of states with denominator 3 observed in the first three subbands of the $n = 2$ LL in bilayer graphene do not exhibit the particle-hole symmetry known from the LLL at $\nu = \frac{1}{3}$ and $\frac{2}{3}$. In higher LLs (including those in experiment presented in Ref. 30), such pairs are not of CF type and correspond to single-loop braid orbits that match the nearest neighbours (at $x + \frac{1}{3}$, where x is a number of the LL subband) and the next-nearest neighbours (at $x + \frac{2}{3}$), both being of electron type. This is also supported by the observation that these pairs are actually asymmetric in R_{xx} response, probably due to higher residual resistivity of the states $x + \frac{2}{3}$ caused by the scattering of uncorrelated nearest neighbours in these states (cf. Figs. 6 and 7 of the main text).

Appendices

A Cyclotron braid commensurability for FQHE states in the LLL in conventional 2DEG

One can identify the correlated states at fractional fillings, generalizing the genuine pattern of the correlation of IQHE, $\frac{S}{N}=\frac{S}{N_0}$, when the cyclotron orbit size $\frac{S}{N_0}$ (where $N_0=\frac{eBS}{hc}$ is the LL degeneracy) fits the electron separation $\frac{S}{N}$. At fractional fillings of the LLL, the cyclotron orbits $\frac{hc}{eB}$ are smaller than $\frac{S}{N}$, and cyclotron orbits cannot match neighbouring particles. For establishing any correlated state, the particle exchanges are, however, necessary to define the statistics of the quantum particles. Exclusively in 2D, multi-loop cyclotron orbits have larger size in comparison to single-loop ones at the same magnetic fields[31,32]. This follows from the distribution of the external field B flux per particle among all loops of the multi-loop cyclotron orbit, which are all located in the same plane. Thus, the condition for commensurability attains the more general form

$$\frac{BS}{N}=(q-1)\frac{hc}{ex}\pm\frac{hc}{ey}, \tag{5}$$

where q is the number of loops of the single cyclotron orbit (q must be an odd integer to ensure that the corresponding braid describes the particle exchange—the braid generator with n additional loops corresponds to a $2n+1=q$-loop cyclotron orbit[12,31]). In magnetic fields in 2D, the braids are built from half-pieces of cyclotron orbits, provided that these orbits accurately fit the neighbouring particle separation at the uniform particle distribution caused by the electric repulsion. In condition (5), $x\geq 1$ (integer) indicates the commensurability of $q-1$ single loops from the q-loop cyclotron orbit to every xth particle on the plane; $y\geq x$ (also integer) indicates the commensurability of the last loop of the q-loop orbit with every yth particle; $\pm$ indicates the same or opposite orientation (of figure-eight shape) of the last, i.e., qth, loop. From (5), we obtain the following conditions:

$$\begin{array}{l}\nu=\frac{N}{N_0}=\frac{xy}{(q-1)y\pm x},\ \text{for LL band electrons,}\\ \nu=1-\frac{xy}{(q-1)y\pm x},\ \text{for LL band holes,}\end{array} \tag{6}$$

for the general hierarchy of correlated states in the LLL describing the FQHE hierarchy. For $x=1$, the hierarchy (6) reproduces the CF hierarchy. For $x>1$, the hierarchy (6) is beyond the ability of the CF model and displays filling ratios for FQHE in the LLL, including those outside the CF hierarchy that were observed in experiment in conventional 2DEG[5]. The comparison with the experimental data is summarized in Fig. 7.

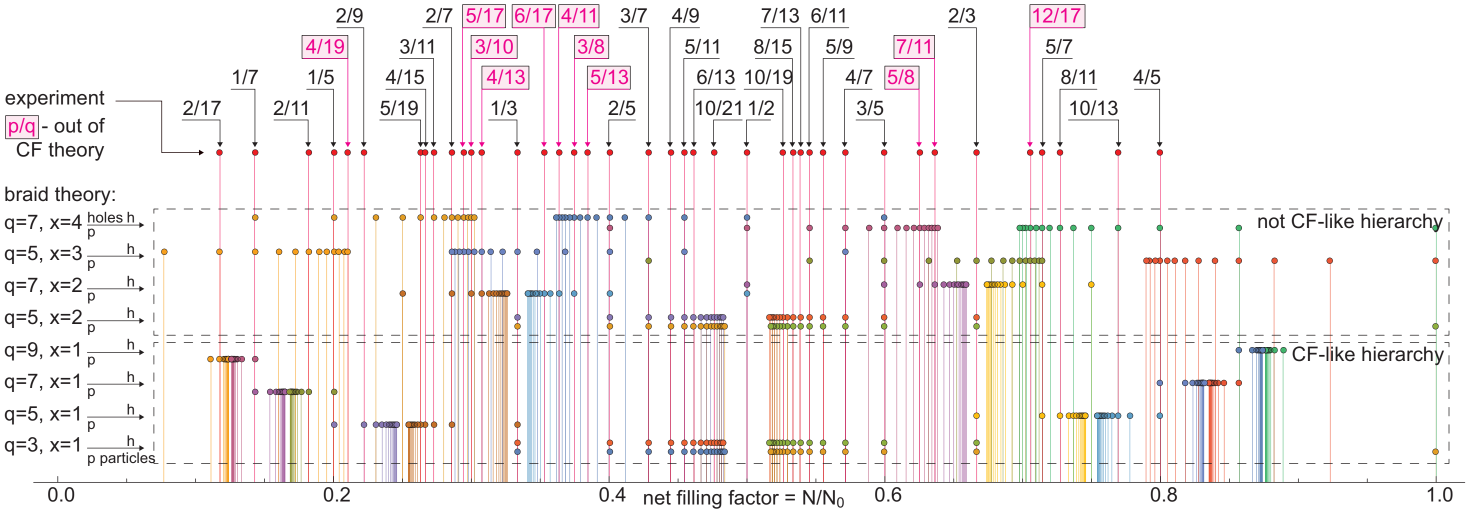

Figure 7. Comparison of the hierarchy (6) with all measured fractional filling rates for FQHE features in the LLL (spin polarized). The hierarchy series (6) for several y are displayed, and filling rates beyond the CF hierarchy are shown in red (the Hall metal state fraction 1/2 is marked).

The CF model agrees with the simplest commensurability case ($x=1$) and breaks down in more complicated commensurability instances, as given by Eq. (6), for $x>1$. In Fig. 7, in red colour, filling rates beyond the main CF hierarchy are indicated, which are observable in experiments and successfully reproduced by the hierarchy (6).

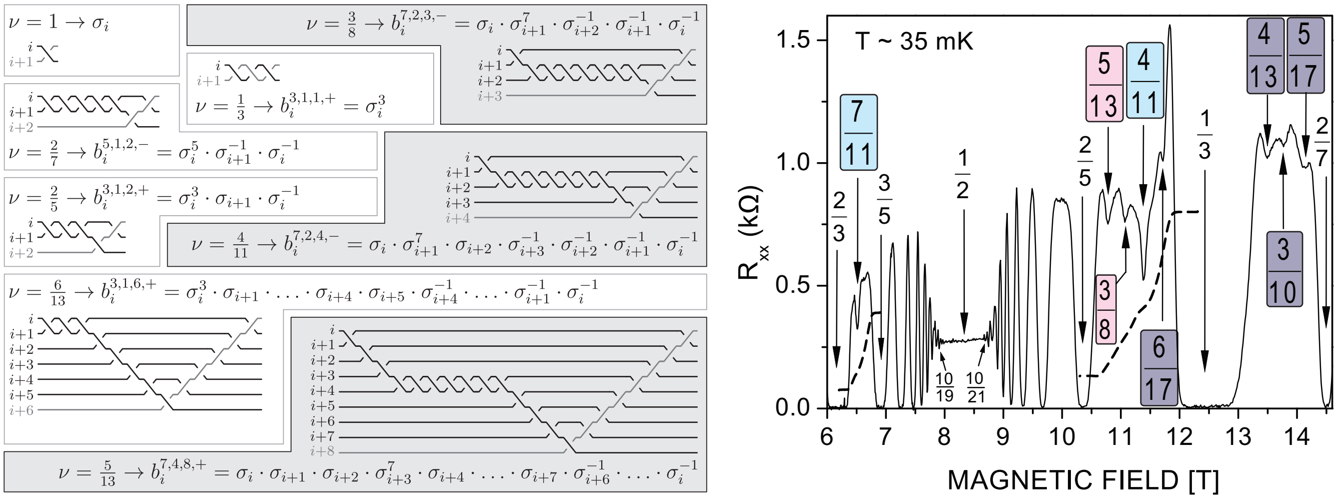

Figure 8. (left) The braid cyclotron subgroup generators for several selected filling fractions (dark background—examples of generators for filling fractions that cannot be derived using standard CF theory, light background—examples of fractions from the Jain-like hierarchy). (right) Measured R_{xx} in the fragment of the LLL in GaAs 2DEG (after Ref. 5), in the same colours as indicated for fractions at which the similar level of R_{xx} is achieved—its nonzero value indicates that not all electrons are involved in the correlated state, as for correlations given by (6) with $x > 1$, i.e., for next-nearest neighbours.

The limit $y \to \infty$ displays the hierarchy of the Hall metal exactly in the same manner as for the archetype of the Hall metal at $\nu = 1/2$ (the last orbit is infinite and fits infinitely distant particles, as in the normal Fermi liquid without any magnetic field[33]). The general Hall metal hierarchy in the LLL thus has the form

$$\begin{array}{l} \nu = \frac{x}{q-1}, \text{ for LL band electrons,} \\ \nu = 1 - \frac{x}{q-1}, \text{ for LL band holes.} \end{array} \tag{7}$$

Note that the Hall metal correlation can be manifested at fractions that do not necessarily have even denominators (for x even, beyond the CF concept), similarly to the hierarchy in (6), which displays fractions with odd and even denominators, in agreement with the experimental observations[5]. Some fractions are repeated in various lines of the general hierarchy (6). This reveals the possibility of various types of commensurability of multi-loop cyclotron orbits with interparticle spacing $\frac{S}{N}$. The advantage of one commensurability over the others (alternative ones at the same filling ratio) is related to energy minimization, i.e., with the minimization of the Coulomb interaction.

B Trial wave functions for FQHE states in the LLL in conventional 2DEG

For the simplest line of the hierarchy (6) with $x = y = 1$, i.e., $\nu = \frac{1}{q}$, where q is an odd integer, the corresponding wave function has been given by Laughlin in the form[34]:

$$\Psi_q(z_1, z_2, \ldots, z_N) = A \prod_{i,j,i>j}^{N,N} (z_i - z_j)^q e^{-\sum_i^N \frac{|z_i|^2}{4l_B^2}}, \tag{8}$$

where $z_i = x_i + iy_i$ is the classical position of the ith particle on the complex plane (the argument of the quantum multiparticle wave function), $l_B = \sqrt{\frac{\hbar c}{eB}}$ is the magnetic length, the product $\prod_{i,j,i>j}^{N,N} (z_i - z_j)^q$ is the Jastrow polynomial, and A is an appropriate normalization constant. The defining characteristic of the Laughlin function is that the q-fold zero at each particle keeps particles apart, and thus diminishes the Coulomb interaction energy. The function (8) must transform itself according to the one unitary representation (1DUR) of the cyclotron braid subgroup with generators σ_i^q. Indeed, for the 1DUR of the full braid group given by $\sigma_i \to e^{i\alpha}$ with $\alpha = \pi$ (fermionic), one gets $e^{iq\pi}$ as the 1DUR of σ_i^q, which coincides with the Laughlin phase.

For the hierarchy (6), the generators (which describe elementary exchanges) of the appropriate more-complicated cyclotron

braid subgroups are defined as follows (for $\pm$ in (6)):

$$
\begin{aligned}
&b_i^{q,x,y,+} = (\sigma_i\cdot\sigma_{i+1}\cdot\ldots\cdot\sigma_{i+x-2}\cdot\sigma_{i+x-1}\cdot\sigma_{i+x-2}^{-1}\cdot\ldots\cdot\sigma_{i+1}^{-1}\cdot\sigma_i^{-1})^{q-1}\cdot\sigma_i\cdot\sigma_{i+1}\cdot\ldots\cdot\sigma_{i+y-2}\cdot\sigma_{i+y-1}\cdot\sigma_{i+y-2}^{-1}\cdot\ldots\cdot\sigma_{i+1}^{-1}\cdot\sigma_i^{-1},\\
&\text{and}\\
&b_i^{q,x,y,-} = (\sigma_i\cdot\sigma_{i+1}\cdot\ldots\cdot\sigma_{i+x-2}\cdot\sigma_{i+x-1}\cdot\sigma_{i+x-2}^{-1}\cdot\ldots\cdot\sigma_{i+1}^{-1}\cdot\sigma_i^{-1})^{q-1}\cdot(\sigma_i\cdot\sigma_{i+1}\cdot\ldots\cdot\sigma_{i+y-2}\cdot\sigma_{i+y-1}\cdot\sigma_{i+y-2}^{-1}\cdot\ldots\cdot\sigma_{i+1}^{-1}\cdot\sigma_i^{-1})^{-1},
\end{aligned}
\tag{9}
$$

with 1DURs (for $\alpha=\pi$) $e^{iq\pi}$ (for $+$) and $e^{i(q-2)\pi}$ (for $-$) (using the following notation for $x(y)=1$: $\sigma_i\cdot\sigma_{i+1}\cdot\dots\cdot\sigma_{i+x-2}\cdot\sigma_{i+x-1}\cdot\sigma_{i+x-2}^{-1}\cdot\dots\cdot\sigma_{i+1}^{-1}\cdot\sigma_i^{-1}=\sigma_i$). Examples of these braid generators are depicted in Fig. 8.

Thus, the related modification of the Jastrow polynomial in the Laughlin function (8) must be as follows (in the LLL, the true wave function must be a holomorphic function that is uniquely defined by its nodes):

$$
\begin{aligned}
\Psi_q^{x,y,+}(z_1,z_2,\ldots,z_N) &= A\prod_{i,j=1;i<i\,mod\,x+(j-1)x}^{N,N/x}(z_i-z_{i\,mod\,x+(j-1)x})^{q-1}\prod_{i,j=1;i<i\,mod\,y+(j-1)y}^{N,N/y}(z_i-z_{i\,mod\,y+(j-1)y})e^{-\sum_i^N\frac{|z_i|^2}{4l_B^2}},\\
\Psi_q^{x,y,-}(z_1,z_2,\ldots,z_N) &= A\prod_{i,j=1;i<i\,mod\,x+(j-1)x}^{N,N/x}(z_i-z_{i\,mod\,x+(j-1)x})^{q-1}\prod_{i,j=1;i<i\,mod\,y+(j-1)y}^{N,N/y}(z_{i\,mod\,y+(j-1)y}-z_i)e^{-\sum_i^N\frac{|z_i|^2}{4l_B^2}}.
\end{aligned}
\tag{10}
$$

The above functions for the CF-like hierarchy ($x=1$) attain the following forms (defined in a unique manner, avoiding a projection from higher LLs onto the LLL in the conventional CF model):

$$
\begin{aligned}
\Psi_q^{x=1,y,+}(z_1,z_2,\ldots,z_N) &= A\prod_{i,j=1,i<j}^{N,N}(z_i-z_j)^{q-1}\prod_{i,j=1;i<i\,mod\,y+(j-1)y}^{N,N/y}(z_i-z_{i\,mod\,y+(j-1)y})e^{-\sum_i^N\frac{|z_i|^2}{4l_B^2}},\\
\Psi_q^{x=1,y,-}(z_1,z_2,\ldots,z_N) &= A\prod_{i,j=1,i<j}^{N,N}(z_i-z_j)^{q-1}\prod_{i,j=1;i<i\,mod\,y+(j-1)y}^{N,N/y}(z_{i\,mod\,y+(j-1)y}-z_i)e^{-\sum_i^N\frac{|z_i|^2}{4l_B^2}}.
\end{aligned}
\tag{11}
$$

The functions (10) are proposed as the trial wave functions for correlated states for filling rates (6), for which elementary exchanges of particles are defined by braids (9), which generalize the Laughlin function (8) for the case when $x,y>1$, with some resemblance to multicomponent Halperin functions[7].

The energy gain in the Laughlin state is due to the lowering of the Coulomb repulsion energy $<\Psi|\sum_{i.j,i>j}^{N,N}\frac{e^2}{|z_i-z_j|}|\Psi>$. The higher x is, the weaker the energy reduction for the function (10) (for the same q and y). This follows from the dilution of correlated particles for $x>1$ (the correlation concerns every xth electron only), as expressed in the modified Laughlin-type function (10), by reducing the domain of the product. This leads to the diminishing of the repulsion energy gain due to the averaging of the Coulomb energy, $\sum_{i.j,i>j}^{N,N}\frac{e^2}{|z_i-z_j|}$, with the wave function (10) instead of (11) (or (8)) because the $q-1$-fold zero in these functions does not prevent approaching all electrons in the case of function (10), but only a fraction $1/x$ (opposite the case of function (8) or (11), for which $x=1$). Therefore, the more stable states are those with lower x. Thus, states with $x=1$ energetically prevail over states with $x>1$ and are more stable. To evaluate the energy values obtained from exact diagonalization for different FQHE fillings[35], the numerical estimation of energy for newly proposed functions (10,11) was performed according to the Monte Carlo Metropolis scheme[36–38]. Some exemplary results, which reveal very good overlap with the exact diagonalization, are presented in Tab. 1.

Nevertheless, it should be noted that from the point of view of the commensurability condition, which governs the form of the cyclotron braid generator corresponding to multi-loop cyclotron orbits, all loops can be featured; thus, each loop can be adjusted to the particle separation independently. Thus, for a q-looped orbit, one would address the ordered series $x_1\leq x_2\leq\cdots\leq x_q$, simplified in (6) to $x_1=\cdots=x_{q-1}=x$, $x_q=y$. Apparently, the Coulomb repulsion minimization prefers $x_1=\cdots=x_{q-1}$, for which the minimization domain restriction (resulting in weaker interaction energy reduction) is more convenient than for distinct distributions of x_i. This explains the choice of the uniform behaviour of the $q-1$ loops (i.e., $x_1=\cdots=x_{q-1}=x$), but this is not a rule and for many fractions, various energetically competitive commensurability opportunities might be considered. This finds a correspondence with the fractal-type character of the FQHE hierarchy as described in Ref. 39.

Let us emphasize that another observation related to various types of correlation identified by the commensurability criterion also agrees with the experimental data for the longitudinal resistivity R_{xx}[5] (Fig. 8 (right)), which is zero for states in which all particles are correlated (i.e., for states with $x=1$), whereas the residual R_{xx} value grows with $x>1$, probably due to the scattering of a portion of the non-correlated electrons, which is in agreement with the presented braid commensurability approach.

q	x	y	hierarchy fraction, $\nu = N/N_0$	energy from Monte Carlo simulation for functions according to Eq. (10,11)	energy from exact diagonalization[35]
3	1	2	$\frac{2\cdot 1}{(3-1)\cdot 2+1} = \frac{2}{5}$	-0.432677	$-0,432804$
3	1	3	$\frac{3\cdot 1}{(3-1)\cdot 3+1} = \frac{3}{7}$	-0.441974	$-0,442281$
3	1	4	$\frac{4\cdot 1}{(3-1)\cdot 4+1} = \frac{4}{9}$	-0.446474	$-0,447442$
3	1	5	$\frac{5\cdot 1}{(3-1)\cdot 5+1} = \frac{5}{11}$	-0.451056	$-0,450797$
5	1	2	$\frac{2\cdot 1}{(5-1)\cdot 2+1} = \frac{2}{9}$	-0.342379	$-0,342742$
5	1	3	$\frac{3\cdot 1}{(5-1)\cdot 3+1} = \frac{3}{13}$	-0.348134	$-0,348349$
5	1	4	$\frac{4\cdot 1}{(5-1)\cdot 4+1} = \frac{4}{17}$	-0.351857	$-0,351189$

Table 1. Comparison of energy values obtained by exact diagonalization and by Monte Carlo simulation for some exemplary filling fractions for FQHE (Monte Carlo Metropolis simulation for the proposed topology-based wave functions, for 200 particles).

References

1. Goerbig, M. O. Electronic properties of graphene in a strong magnetic field. *Rev. Mod. Phys.* **83**, 1193 (2011).
2. McCann, E. & Koshino, M. The electronic properties of bilayer graphene. *Rep. Prog. Phys.* **76**, 056503 (2013).
3. Papic, Z. & Abanin, D. A. Topological phases in the zeroth Landau level of bilayer graphene. *Phys. Rev. Lett.* **112**, 046602 (2014).
4. Jain, J. K. *Composite Fermions* (Cambridge UP, Cambridge, 2007).
5. Pan, W. *et al.* Fractional quantum Hall effect of composite fermions. *Phys. Rev. Lett.* **90**, 016801 (2003).
6. Mukherjee, S., Mandal, S. S., Wu, Y., Wójs, A. & Jain, J. K. Enigmatic 4/11 state: A prototype for unconventional fractional quantum Hall effect. *Phys. Rev. Lett.* **112**, 016801 (2014).
7. Halperin, B. I. Theory of the quantized Hall conductance. *Helv. Phys. Acta* **56**, 75 (1983).
8. Wu, Y. S. General theory for quantum statistics in two dimensions. *Phys. Rev. Lett.* **52**, 2103 (1984).
9. Wilczek, F. *Fractional Statistics and Anyon Superconductivity* (World Sc., Singapore, 1990).
10. Laidlaw, M. G. & DeWitt, C. M. Feynman functional integrals for systems of indistinguishable particles. *Phys. Rev. D* **3**, 1375 (1971).
11. Birman, J. S. *Braids, Links and Mapping Class Groups* (Princeton UP, Princeton, 1974).
12. Jacak, J., Gonczarek, R., Jacak, L. & Jóźwiak, I. *Application of Braid Groups in 2D Hall System Physics: Composite Fermion Structure* (World Scientific, 2012).
13. Jacak, J. & Jacak, L. Recovery of Laughlin correlations with cyclotron braids. *EPL* **92**, 60002 (2010).
14. Imbo, T. D., Imbo, C. S. & Sudarshan, C. S. Identical particles, exotic statistics and braid groups. *Phys. Lett. B* **234**, 103 (1990).
15. Jacak, J. & Jacak, L. The commensurability condition and fractional quantum Hall effect hierarchy in higher Landau levels. *JETP Lett.* **102**, 19 (2015).
16. Eisenstein, J. P., Lilly, M. P., Cooper, K. B., Pfeiffer, L. N. & West, K. W. New physics in high Landau levels. *Phys. E* **6**, 29 (2000).
17. Dolev, M. *et al.* Characterizing neutral modes of fractional states in the second Landau level. *Phys. Rev. Lett.* **107**, 036805 (2011).
18. Willett, R. L. The quantum Hall effect at 5/2 filling factor. *Rep. Prog. Phys.* **76**, 076501 (2013).
19. Castro Neto, A. H., Guinea, F., Peres, N. M. R., Novoselov, K. S. & Geim, A. K. The electronic properties of graphene. *Rev. Mod. Phys.* **81**, 109 (2009).
20. Amet, F. *et al.* Composite fermions and broken symmetries in graphene. *Nat. Comm.* **6**, 5838 (2014).
21. Ki, D. K., Falko, V. I., Abanin, D. A. & Morpurgo, A. Observation of even denominator fractional quantum Hall effect in suspended bilayer graphene. *Nano Lett.* **14**, 2135 (2014).
22. Kou, A. *et al.* Electron-hole asymmetric integer and fractional quantum Hall effect in bilayer graphene. *Sci.* **345**, 55 (2014).

23. Maher, P. *et al.* Tunable fractional quantum Hall phases in bilayer graphene. *Sci.* **345**, 61 (2014).

24. Kim, Y. *et al.* Fractional quantum Hall states in bilayer graphene probed by transconductance fluctuations. *Nano Lett.* **15**, 7445 (2015).

25. Feldman, B. E., Krauss, B., Smet, J. H. & Yacoby, A. Unconventional sequence of fractional quantum Hall states in suspended graphene. *Sci.* **337**, 1196 (2012).

26. Feldman, B. E. *et al.* Fractional quantum Hall phase transitions and four-flux states in graphene. *Phys. Rev. Lett.* **111**, 076802 (2013).

27. Dean, C. R. *et al.* Multicomponent fractional quantum Hall effect in graphene. *Nat. Phys.* **7**, 693 (2011).

28. Suen, Y. W., Engel, L. W., Santos, M. B., Shayegan, M. & Tsui, D. C. Observation of a ν=1/2 fractional quantum Hall state in a double-layer electron system. *Phys. Rev. Lett.* **68**, 1379 (1992).

29. Eisenstein, J. P., Boebinger, G. S., Pfeiffer, L. N., West, K. W. & He, S. New fractional quantum Hall state in double-layer two-dimensional electron systems. *Phys. Rev. Lett.* **68**, 1383 (1992).

30. Diankov, G. *et al.* Robust fractional quantum Hall effect in the n=2 Landau level in bilayer graphene. *Nat. Comm.* **7**, 13908 (2016).

31. Jacak, J., Jóźwiak, I. & Jacak, L. New implementation of composite fermions in terms of subgroups of a braid group. *Phys. Lett. A* **374**, 346 (2009).

32. Jacak, J., Jóźwiak, I., Jacak, L. & Wieczorek, K. Cyclotron braid group structure for composite fermions. *J. Phys: Cond. Matt.* **22**, 355602 (2010).

33. Stormer, H. L. *et al.* The fractional quantum Hall effect in a new light. *Semicond. Sci. Technol.* **9**, 1853 (1994).

34. Laughlin, R. B. Anomalous quantum Hall effect: an incompressible quantum fluid with fractionally charged excitations. *Phys. Rev. Lett.* **50**, 1395 (1983).

35. Balram, A. C., Töke, C., Wójs, A. & Jain, J. K. Fractional quantum Hall effect in graphene: Quantitative comparison between theory and experiment. *Phys. Rev. B* **92**, 075410 (2015).

36. Ciftja, O. & Wexler, C. Monte Carlo simulation method for Laughlin-like states in a disk geometry. *Phys. Rev. B* **67**, 075304 (2003).

37. Morf, R. & Halperin, B. I. Monte Carlo evaluation of trial wavefunctions for the fractional quantized Hall effect: Spherical geometry. *Z. Phys. B* **68**, 391 (1987).

38. Metropolis, N., Rosenbluth, A. W., Rosenbluth, M. N., Teller, A. M. & Teller, E. Equation of state calculations by fast computing machines. *J. Chem. Phys.* **21**, 1087 (1953).

39. Mani, R. G. & von Klitzing, K. Fractional quantum Hall effects as an example of fractal geometry in nature. *Z. Phys. B* **100**, 635 (1996).
